# Differentiation and activation of human CD4 T cells is associated with a gradual loss of myelin and lymphocyte protein

**DOI:** 10.1002/eji.202048603

**Published:** 2021-01-25

**Authors:** Judith Leitner, Kodchakorn Mahasongkram, Philipp Schatzlmaier, Karin Pfisterer, Vladimir Leksa, Supansa Pata, Watchara Kasinrerk, Hannes Stockinger, Peter Steinberger

**Affiliations:** ^1^ Division of Immune Receptors and T Cell Activation Institute of Immunology Center for Pathophysiology, Infectiology and Immunology Medical University of Vienna Vienna Austria; ^2^ Division of Clinical Immunology Department of Medical Technology Faculty of Associated Medical Sciences Chiang Mai University Chiang Mai Thailand; ^3^ Institute for Hygiene and Applied Immunology, Centre for Pathophysiology, Infectiology and Immunology Medical University of Vienna Vienna Austria; ^4^ Department of Dermatology Medical University of Vienna Vienna Austria; ^5^ Laboratory of Molecular Immunology Institute of Molecular Biology Slovak Academy of Sciences Bratislava Slovakia; ^6^ Biomedical Technology Research Centre National Centre for Genetic Engineering and Biotechnology National Science and Technology Development Agency at the Faculty of Associated Medical Sciences Chiang Mai University Chiang Mai Thailand

**Keywords:** CD4, Differentiation, Human, MAL, T‐cell activation

## Abstract

Upon generation of monoclonal antibodies to the T cell antigen receptor/CD3 (TCR/CD3) complex, we isolated mAb MT3, whose reactivity correlates inversely with the production of IFN‐γ by human peripheral blood T lymphocytes. Using eukaryotic expression cloning, we identified the MT3 antigen as myelin‐and‐lymphocyte (MAL) protein. Flow cytometry analysis demonstrates high surface expression of MAL on all naïve CD4^+^ T cells whereas MAL expression is diminished on central memory‐ and almost lost on effector memory T cells. MAL^–^ T cells proliferate strongly in response to stimulation with CD3/CD28 antibodies, corroborating that MAL^+^ T cells are naïve and MAL^–^ T cells memory subtypes. Further, resting MAL^–^ T cells harbor a larger pool of Ser59‐ and Tyr394‐ double phosphorylated lymphocyte‐specific kinase (Lck), which is rapidly increased upon in vitro restimulation. Previously, lack of MAL was reported to prevent transport of Lck, the key protein tyrosine kinase of TCR/CD3 signaling to the cell membrane, and to result in strongly impaired human T cell activation. Here, we show that knocking out MAL did not significantly affect Lck membrane localization and immune synapse recruitment, or transcriptional T cell activation. Collectively, our results indicate that loss of MAL is associated with activation‐induced differentiation of human T cells but not with impaired membrane localization of Lck or TCR signaling capacity.

## Graphical abstract

Myelin‐and‐lymphocyte protein isoform‐A (MAL‐A) is highly expressed on human naive CD4^+^ T cells. Activation‐induced proliferation and differentiation is associated with a gradual loss of MAL expression, resulting in highly signalling‐competent effector‐memory cells with enhanced IFN‐γ‐secretion. Knockout of MAL does not affect recruitment of lymphocyte‐specific kinase Lck and TCR downstream signalling.

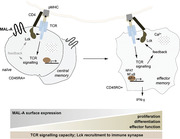

## Introduction

The cognate interaction of the T‐cell antigen receptor/CD3 complex (TCR/CD3) with peptide‐MHC complexes displayed on APC is the first step of a complex and multilayered process that can either lead to a productive response or to a state of unresponsiveness in T cells. Upon engagement, the TCR/CD3—in conjunction with Lck (lymphocyte‐specific kinase)—initiates a tyrosine phosphorylation cascade that triggers distinct signaling pathways [1, 2, 3, 4]. Its functional outcome, namely transcriptional reprogramming, proliferation, and differentiation of T cells is orchestrated by the formation of a stable as well as dynamic immunological synapse (IS) [5, 6, 7]. It is thought that the IS endows T cells with an exquisite ability to discriminate between antigenic ligands of high and low affinity, thereby contributing to T‐cell activation decisions. Notably, the activation threshold and the quality of responses profoundly change as naïve T cells differentiate toward effector cells [8, 9]. A plethora of surface molecules and signaling compounds take part in TCR/CD3‐induced signaling complexes and IS formation, and intense efforts have been made to identify and characterize these molecules. Nevertheless, there are controversies for many molecules regarding their precise role during T‐cell responses, and it is likely that there are still undefined molecules that take part in T‐cell signaling processes.

In an effort to characterize such molecules, we have previously used CD3 monoclonal antibody (mAb)‐coated beads to precipitate TCR/CD3 complexes from human PBMC lysates. Mice were repeatedly immunized with the resulting precipitates and, using standard hybridoma techniques, a mAb was generated from splenocytes of the immunized animals [10]. This mAb termed MT3 was shown to react with subsets of CD4^+^ and CD8^+^ T cells, whereas binding to CD19^+^ B cells or CD56^+^ NK cells was not detected. Moreover, we found that the MT3 antigen colocalized with the TCR/CD3 complex on the surface of the Jurkat T‐cell line and peripheral human T cells [10].

Here, we show that mAb MT3 reacts with T cells that do not produce IFN‐γ upon short‐term stimulation with PMA/ionomycin. By eukaryotic expression cloning, we identified the MT3 antigen as the myelin and lymphocyte protein (MAL). MAL is an integral membrane protein containing a MARVEL (MAL and related proteins for vesicle trafficking and membrane link) domain [11]. We show that the mAb MT3 reacts with MAL‐A, the large isoform of MAL, and demonstrate that this is the predominantly expressed form in human T cells and T‐cell lines. Using multicolor flow cytometry, we found that MAL surface expression is gradually lost during the differentiation and activation of CD4^+^ T cells. Upon CD3/CD28 stimulation, lack of surface MAL correlated with robust T‐cell proliferation, high production of IFN‐γ, and enhanced feedback‐induced Lck phosphosignature. MAL was previously reported to have an essential role in targeting the Src family kinase Lck to plasma membrane rafts of human T cells. The transport of Lck is indispensable for synapse formation and proper T‐cell activation [12]. However, when we used CRISPR‐Cas9 to delete MAL in a triple reporter T‐cell line based on human Jurkat cells, the loss of MAL was not associated with membrane localization of Lck and impaired activation of the transcription factors NF‐κB, NFAT, and AP‐1, which are downstream of Lck signaling.

## Results

### MT3 expression defines T cells that lack IFN‐γ production upon PMA/ionomycin stimulation

We have previously described mAb MT3 recognizing a molecule associated with the TCR/CD3 complex. The mAb was generated after immunizing mice with CD3‐coprecipitated complexes obtained from PBMC lysates. The specificity of mAb MT3 with the TCR/CD3 complex was confirmed by confocal microscopy and flow cytometry, and revealed that MT3 marked a subpopulation of human T cells [10].

**Figure 1:**
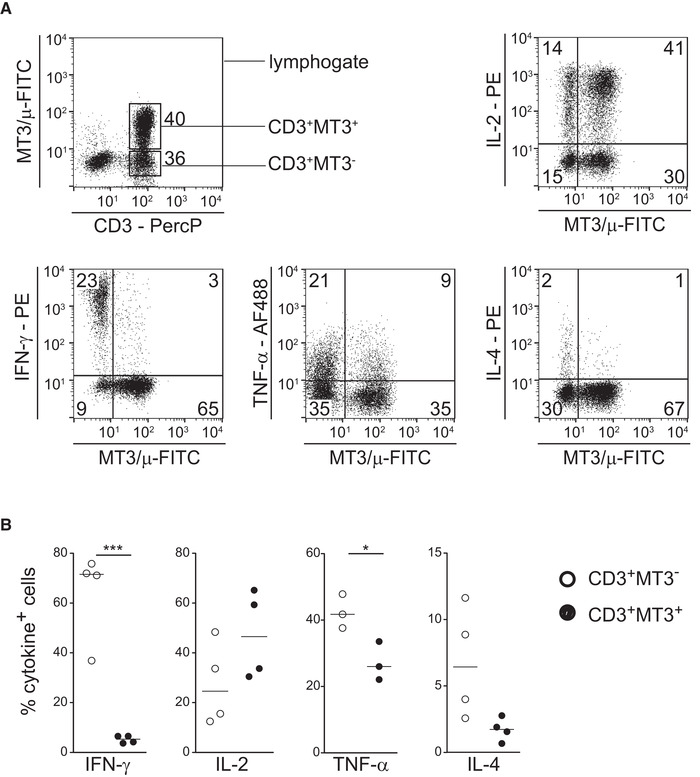
MT3^–^ T cells are rapid producers of IFN‐γ. Human PBMCs were stimulated with PMA plus ionomycin for 6 h in the presence of monensin and stained for surface‐expressed CD3 and MT3 plus intracellular IFN‐γ, TNF‐α, IL‐2, or IL‐4 and measured by flow cytometry. (A) Dot plots showing the surface expression of the MT3 antigen among lymphocytes (top left panel). Then, CD3^+^ T‐cells were gated and analyzed for coexpression of MT3 and cytokines. Frequencies of cells per quadrant are given in % for one representative donor. (B) Percentage of cytokine producing cells in MT3^–^ and MT3^+^ CD3^+^ T cells and median of three (TNF‐α) or four donors (other cytokines) are shown. Each dot represents one donor. Data are from three experiments with 1‐2 donors; n = 3 for TNF‐α; n = 4 for IFN‐γ, IL‐2, IL‐4; *p < 0.05; ***p < 0.001, two‐sided paired *t*‐test).

To characterize the MT3^+^ T‐cell subset(s), we activated human PBMCs for 6 h with PMA plus ionomycin and analyzed expression of the cytokines IFN‐γ, IL‐2, IL‐4, and TNF‐α together with MT3 surface expression. The gating strategy is displayed in Supporting Information Fig. S1A. This short‐term in vitro stimulation did not significantly alter the MT3 surface profile itself (Supporting information Fig. S1B). The MT3^–^T cells produced significantly more IFN‐γ and TNF‐α than the MT3^+^ T cells. The percentage of IL‐4 producing cells was also higher in the MT3^–^ subset but this difference did not reach statistical significance. In contrast, the majority of the MT3^+^ T cells were IL‐2 producers (Fig. 1).

### Molecular cloning of the MT3 antigen

**Figure 2:**
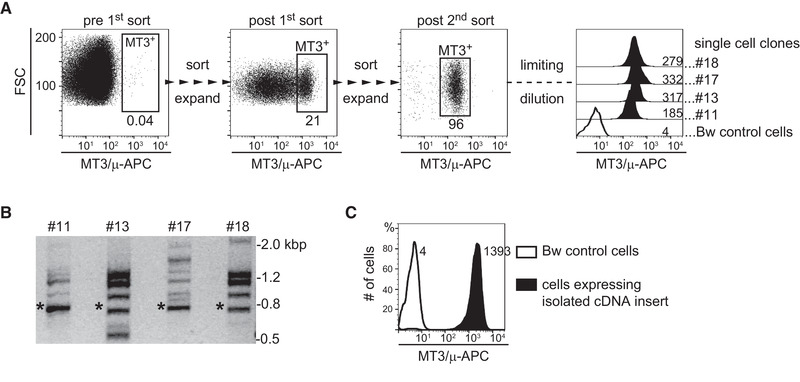
Molecular cloning of the MT3 antigen. (A) Bw cells transduced with cDNA libraries of human T cells and human DCs were stained for expression of the MT3 antigen and reactive cells were enriched by two rounds of cell sorting. Single‐cell clones (SCCs) were established by limiting dilution and analyzed for MT3 surface expression (right panel). The geometric MFI (gMFI) value is shown for each histogram. (B) PCR amplification of retroviral cDNA inserts of genomic DNA isolated from MT3^+^ clones retrieved a 0.7 kb insert present in all SCCs (indicated by an asterisk). (A,B) Data for four clones (n = 4) are shown. (C) The 0.7 kb cDNA was isolated, cloned in a retroviral expression vector and re‐expressed in Bw cells. Transduced cells (filled histogram) and control‐transduced cells (open histogram) were probed with MT3 antibody and measured by flow cytometry (gMFI values are shown). Identity of the MT3 antigen was confirmed in three independent experiments.

To identify the antigen recognized by mAb MT3, mouse thymoma Bw5147 cells (short designation within this work Bw cells) expressing retroviral cDNA libraries of human T cells and DCs [13, 14, 15] were subjected to two rounds of sorting upon surface staining with mAb MT3. Cell clones established from the MT3^+^ population were tested for expression of the MT3 antigen (Fig. 2A). Genomic DNA was prepared from the MT3^+^ clones and retroviral cDNA inserts were PCR retrieved using primers specific for the flanking retroviral sequences. A band of approximately 0.7 kb was present in all MT3^+^ clones tested (Fig. 2B). The band was isolated, cloned, and re‐expressed in Bw cells. The resulting Bw cells strongly reacted with mAb MT3 (Fig. 2C). Subsequently, the 0.7 kb band was sequenced and subjected to BLAST analysis. The obtained DNA sequence was identical with cDNAs encoding human MAL.

**Figure 3:**
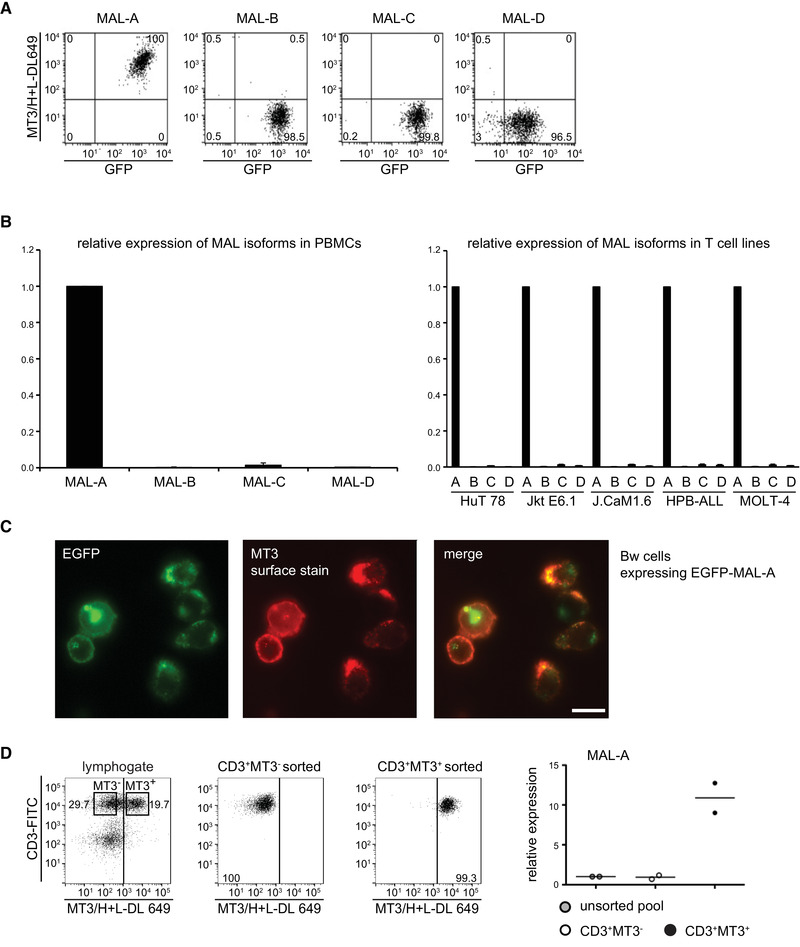
The mAb MT3 recognizes MAL‐A, the major MAL isoform in T cells. (A) Bw cells coexpressing either the MAL‐A, ‐B, ‐C, or ‐D splice variant plus eGFP from bicistronic retroviral vectors were probed with mAb MT3 and analyzed by flow cytometry. Frequencies of cells per quadrant are given in %. Data shown are representative for three independently performed experiments (n = 3). (B) cDNAs derived from human PBMCs and T‐cell lines were subjected to qPCR using primer pairs specific for the different MAL splice variants. Mean ± SD of triplicate measurements are shown. Data shown is representative for three independently performed experiments (n = 3). (C) Bw cells expressing GFP‐tagged MAL‐A were surface‐stained with MT3 followed by DyLight‐649 (DL 649) labeled secondary antibody. Colocalization of the GFP and DyLight‐649 signal (depicting surface‐resident GFP‐MAL) was analyzed via fluorescence microscopy. Experiment is representative for two independently performed (n = 2). Scale bar, 20 μm. (D) MT3 surface positive and negative CD3^+^ T cells were flow sorted from PBMCs with sorting gates indicated (left panel). Purity of the sorted populations was assessed by reanalysis of sorted cells (second and third panel). Frequencies of cells per quadrant are given in %. cDNA derived from unsorted PBMCs, MAL^–^ or MAL^+^ sorted T cells was analyzed for MAL‐A expression by qPCR (right panel). For standardization of gene expression GAPDH was used as an endogenous control. The mRNA target gene expression levels were normalized to the endogenous control and expressed in relationship to the expression levels in the control cells (unsorted pool) as 2^−▵▵CT^ (Livak Method). Data show mean of duplicate measurements from one donor and are representative for two donors.

MAL is an integral membrane protein with a four transmembrane‐helix architecture and both the *N*‐ and *C*‐terminal regions are located in the cytoplasm [16]. Four isoforms (MAL‐A, MAL‐B, MAL‐C, and MAL‐D) arise from alternative splicing of the *MAL* gene encompassing four exons [17]. Sequence analysis revealed that the MT3 antigen corresponds to MAL‐A (NM_002371), the largest isoform of MAL. To analyze whether the MT3 antibody also bound to other isoforms, a bicistronic vector was employed to coexpress cDNAs encoding MAL splicing variants with eGFP. Bw cells transduced with the different constructs were all positive for GFP, whereas only the construct encoding MAL‐A conferred reactivity upon surface staining with mAb MT3 (Fig. 3A).

Next, we generated four primer pairs to specifically amplify MAL‐variants to assess their expression in various cell types. We found that MAL‐A was the major form of MAL expressed by human PBMCs and human T‐cell lines (Fig. 3B). Further, we generated and expressed an eGFP‐MAL fusion protein in Bw cells. Microscopic analysis revealed that a large fraction of eGFP‐MAL was surface resident and colocalized with MT3 (Fig. 3C). However, the chimeric eGFP‐MAL protein was also detected in distinct intracellular compartments. Flow cytometric analysis of human primary T cells revealed a significant proportion of T cells that did not express the MT3 antigen on their surface (Fig. 1). Therefore, we asked whether this subpopulation expressed MAL intracellularly. Since the IgM mAb MT3 could not be used for intracellular staining (our unpublished observation), we assessed total MAL‐A expression by RT‐qPCR. cDNAs derived from MAL^+^ and MAL^–^ human T cells sorted from PBMCs were subjected to RT‐qPCR. Some MAL mRNA expression was detected also in MAL^–^ T cells, although the expression levels were much higher in MAL^+^ cells (Fig. 3D). Collectively, these results indicate that MT3 recognizes the splicing variant MAL‐A on the surface of human T cells.

### MAL is downregulated during in vitro activation of human CD4 T cells

**Figure 4:**
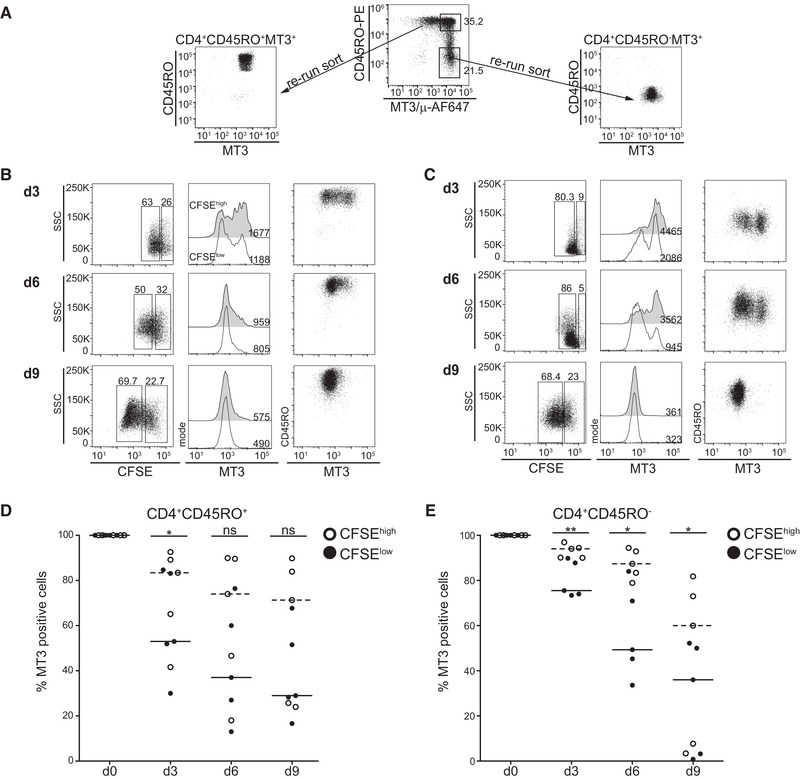
MAL surface expression characterizes naïve and nonproliferating T‐cell subsets. (A) CD4^+^CD45RO^+^MT3^+^ (memory) and CD4^+^CD45RO^–^MT3^+^ (naïve) cells were sorted and the purity of the sorted population was confirmed by flow cytometry. A representative experiment is shown. (B, C) Isolated cells were CFSE‐labeled and stimulated with immobilized CD3 and CD28 mAbs. At the indicated time points cells were harvested, stained for CD45RO and MAL surface expression and analyzed by flow cytometry. Flow cytometry analysis of MT3^+^CD4 memory T cells (B) and naïve MT3^+^CD4 T cells (C) was performed. Left panels: CFSE‐dilution and gating on CFSE^high^ and CFSE^low^ cells is shown; middle panels: CFSE^high^ (filled histograms) and CFSE^low^ (open histograms) were analyzed for expression of the MT3 antigen; right panels: Dot plots showing CD45RO and MT3 expression. For all histograms, gMFI values are given. (D, E) Summarized data of five individual donors showing percentages of MT3^+^ cells in the CFSE^high^ and CFSE^low^ populations for sorted MT3^+^memory (D) or MT3^+^naïve (E) CD4 T cells after in vitro stimulation for the indicated time points. (n = 3, three experiments with 1‐2 donors per experiment, dashed lines median value for CFSE^high^ T cells; solid line median value for CFSE^low^ T cells). **p < 0.001, *p < 0.01, ns…not significant, two‐sided paired *t*‐test.

To analyze the regulation of MAL during in vitro activation of human CD4^+^ T cells, MAL expressing naïve T cells (CD45RO^–^) and memory T cells (CD45RO^+^) were isolated by flow sorting (Fig. 4A). Cells were CFSE labeled and following 3, 6, and 9 days of stimulation cells were harvested, stained for expression of MAL and CD45RO, and analysed by flow cytometry. The detailed gating strategy is shown in Supporting Information Fig. S2A and B. Results of naïve and memory in vitro stimulated CD4^+^ T cells of a representative donor are shown in Fig. 4B and C, respectively. In both subsets, a loss of MAL expression was observed and cells that had proliferated had lower expression than their nonproliferated counterparts. Analysis of CD4^+^ T cells of five donors demonstrated a continuous loss of MAL expression in the course of the stimulation experiments. In the proliferated (CFSElow) cells, the loss of MAL was more pronounced (Fig. 4D and E). No upregulation of MAL was detected in sorted MAL‐negative CD4^+^ T cells upon in vitro stimulation (Supporting information Fig. S2C).

**Figure 5:**
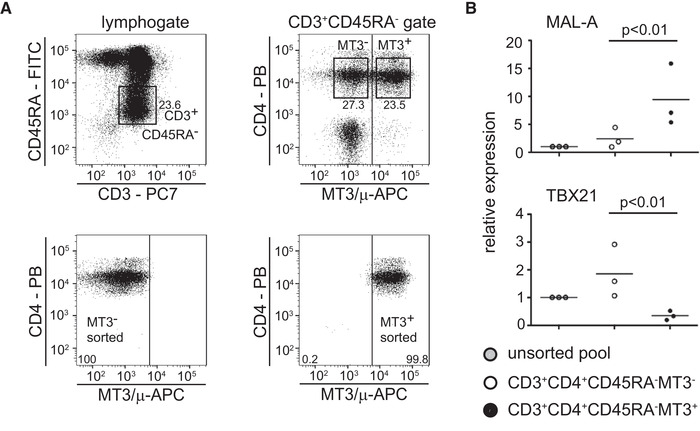
TXB21 is preferentially expressed in CD4^+^ MAL^–^ T cells. (A) CD3^+^CD4^+^CD45RA^–^ T cells were separated from PBMCs into MT3^–^ and MT3^+^ cells by flow sorting as indicated (top panels); the purity of the sorted populations was reassessed by flow cytometry (bottom panels). Numbers indicate percentages of cells (in the gates). Data are representative for three independently performed experiments (n = 3, one donor per experiment). (B) Expression of MAL‐A and TBX21 in unsorted PBMCs and in the MT3^–^ and MT3^+^CD4^+^CD45RA^–^ T cells was analyzed by qPCR. Data show mean of duplicate measurement of three different donors (n = 3). Each donor was analyzed in an independent experiment. For statistics, paired *t*‐test was used.

In a next set of experiments, we analyzed whether transcription factors specifying distinct T‐cell subsets were differentially expressed in MAL^+^ and MAL^–^ T cells. We focused on CD45RA^–^CD4^+^ non‐naïve T cells, since our previous results indicated that this subset contained comparable numbers of MAL^+^ and MAL^–^ T‐cells [10]. Highly pure MAL^+^ and MAL^–^ CD45RA^–^CD4^+^ T‐cells were isolated by FACS using the gating strategy indicated in Fig. 5A. Isolated cells were immediately subjected to RNA isolation and RT‐qPCR analysis. We did not observe significant differences in the expression of GATA3, FOXP3, AHR, BCL6, cMAF, RunX3, and PU.1 (Supporting information Fig. S3).However, we observed a significantly higher TBX21 expression in the MAL^–^ subset (Fig. 5B).

### MAL is preferentially expressed on naïve and central memory CD4 T cells

**Figure 6:**
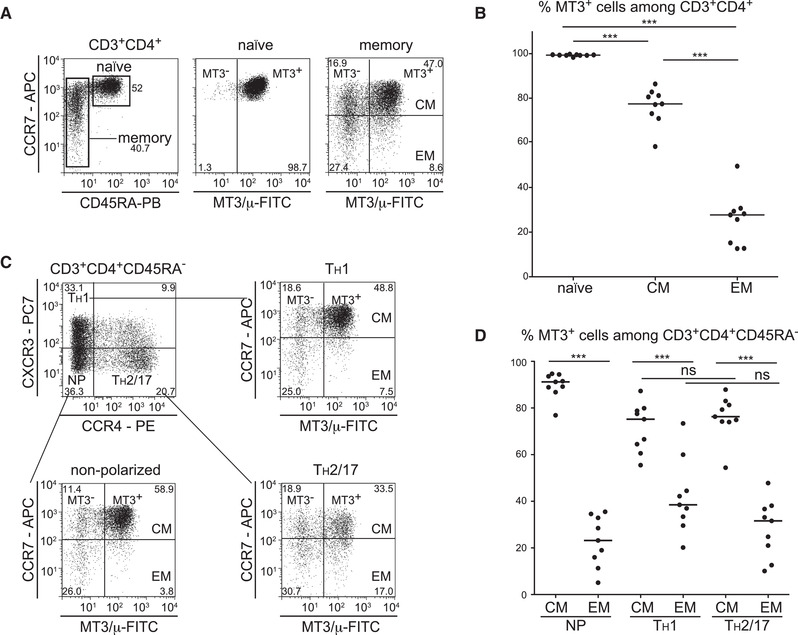
Differentiation from naïve to central and effector memory CD4^+^ T cells is associated with progressive loss of MAL surface expression. For multicolor flow cytometry analysis, human PBMCs were costained with mAbs to CD3, CD4, CD45RA, CCR7, CXCR3, CCR4, and MT3. (A) Gating strategy to identify naïve and memory CD4 T cells and MAL expression on these subsets is shown. Frequencies of cells per gate are given in %. (B) Percentage of MT3^+^ CD4^+^ T cells in naïve, central memory (CM) and effector memory (EM) cells of healthy donors (n = 9). Each dot represents the percentage of MT3^+^ CD4^+^ T cells of one donor in the indicated subset, the median is shown as dashed line. (C) Gating strategy to identify nonpolarized (NP), Th1 and Th2/17 cells in the CM and EM CD4^+^ T cells is shown. Frequencies of cells per gate are given in %. (D) Summary of all donors analyzed (n = 9) is shown. Each dot represents the percentage of MT3^+^ CD4^+^ T cells of one donor, the median is indicated. (A, C) Data shown are representative for nine donors. (B, D) Summarized data of three experiments with three donors each. Nine different donors were analyzed. One‐way ANOVA followed by Tukey's multiple comparison test was used for statistical evaluation (***p < 0.001; ns, not significant).

To gain a better insight into the surface expression of MAL on human T‐cell subsets, we performed multicolour flow cytometric analysis using CD4^+^ peripheral blood T cells derived from healthy volunteers. Freshly isolated PBMCs were stained with mAb MT3 together with mAbs against CD3, CD4, CD45RA, and CCR7 to discriminate naïve (CD45RA^+^), central memory (CM) (CD45RA^–^ CCR7^+^), and effector memory (EM) (CD45RA^–^CCR7^–^) CD4^+^ T cells. The detailed gating strategy is given in Supporting Information Fig. S4. MAL was uniformly expressed on naïve CD4^+^ T cells; the majority of CM T cells also expressed MAL. In contrast, only a small subset of EM T cells was MAL^+^ (Fig. 6A). Analysis of CD4^+^ T cells of nine donors confirmed that the differentiation of CD4^+^ T cells from the naïve to the CM and the EM state was associated with a gradual loss of MAL surface expression (Fig. 6B).

Because qPCR analysis showed that in memory CD4^+^ T cells the expression of TBX21 encoding T‐bet, the master regulator of Th1 T cells, was inversely correlated with expression of MAL, we assessed whether MAL might be differentially expressed in Th1 and Th2 cells. We performed seven‐color flow cytometry by staining PBMCs with mAbs to CD3, CD4, CD45RA, CCR7, CXCR3, CCR4, and MAL. By using this mAb panel and the gating strategy depicted in Fig. 6C, we were able to further discriminate CM and EM CD4^+^ T cells into Th1 (CXCR3^+^), Th2/Th17 (CCR4^+^), and non‐Th1/Th2 polarized T cells (CXCR3^–^CCR4^–^). Analysis of MAL expression in these six subsets underscored a significant downregulation in surface expression of MAL on EM cells in all Th‐subsets tested (Fig. 6C and D). These results show that independently of the Th‐subset, the majority of CM cells express MAL, whereas most EM cells are MAL‐negative.

### MAL surface expression is associated with reduced feedback phosphosignature of Lck

**Figure 7:**
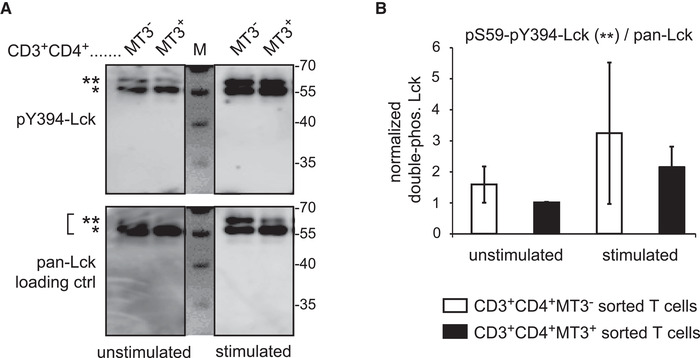
MAL surface expression is associated with less feedback‐induced phospho‐signature of lymphocyte‐specific protein‐tyrosine kinase Lck. (A) CD3^+^CD4^+^ T cells were sorted from PBMCs into MT3^–^ and MT3^+^ cells. Cells were left unstimulated or were stimulated by CD3 mAb OKT3. Total cell lysates were generated for semiquantitative Western blot analysis. Membranes were probed for Y394‐phosphorylated Lck and, after stripping, reprobed for pan‐Lck loading control via specific antibodies as indicated. Shown are Lck‐specific bands at 56 (*) and 59 (**) kDa, the latter indicative of Lck also phosphorylated at S59 by ERK upon TCR induction. M, size marker lane. Data shown are representative for three independently performed experiments. (B) Densitometric analysis of double phosphorylated Lck (pY394‐signals at 59 kDa, **) normalized to pan‐Lck expression. Normalized signal was lowest for unstimulated MT3^+^ cells and set to 1 for each donor. Mean ± SD of three donors analyzed in three independent experiments are shown (n = 3).

The MAL protein was described to be a component of the transport machinery of Lck [12]. Therefore, we analyzed the expression level and the phosphosignature of the lymphocyte‐specific kinase Lck, the 56 kDa key kinase of TCR‐proximal signal transduction. Activity of its kinase is linked to a hallmark activatory phosphorylation at tyrosine 394 (Y394) induced by TCR/CD3 engagement, allowing phosphorylation of CD3zeta by Lck followed by activation of ZAP‐70 and downstream effector molecules, for example, extracellular signal‐regulated kinase ERK1/2 [18]. Activated ERK in turn phosphorylates Lck at serine 59 (S59), a hallmark TCR‐induced feedback signature with ambiguous effects on signaling capacity [19, 20, 21]. Notably, S59 phosphorylation is associated with a shift of Lck migration from 56 to 59 kDa. To investigate Lck phosphorylation, we isolated MAL^–^ and MAL^+^ CD4^+^ T‐cells by sorting as depicted in Supporting information Fig. S5, and generated cell lysates from resting cells as well as upon stimulation using CD3 mAb. By Western blot analysis shown in Fig. 7A, we probed for pY394 phosphorylated Lck and pan‐Lck expression. Resting MAL^‐^ cells harbored a larger pool of size‐shifted S59‐Y394 double phosphorylated Lck (marked by **) compared to MAL^+^ cells. This pool was readily enhanced upon short‐term in vitro stimulation. Normalization of double phosphorylated Lck to pan‐Lck expression levels by densitometric analysis (Fig. 7B) confirmed that MAL surface‐negative cells featured an enlarged pool of double‐phosphorylated Lck.

Upon stimulation, this pool, albeit nonsignificantly, was more rapidly increased in the MAL^–^ compared to the MAL^+^ cells.

### Impact of MAL deficiency on T cell activation

Previous studies have reported that a Jurkat T‐cell mutant, which was isolated by chemical mutagenesis, had defective TCR‐induced signaling (JTIM) and no detectable MAL mRNA. Additionally, the authors described a profound reduction of cell membrane localized Lck in these as well as in cells expressing MAL siRNA [12].

**Figure 8:**
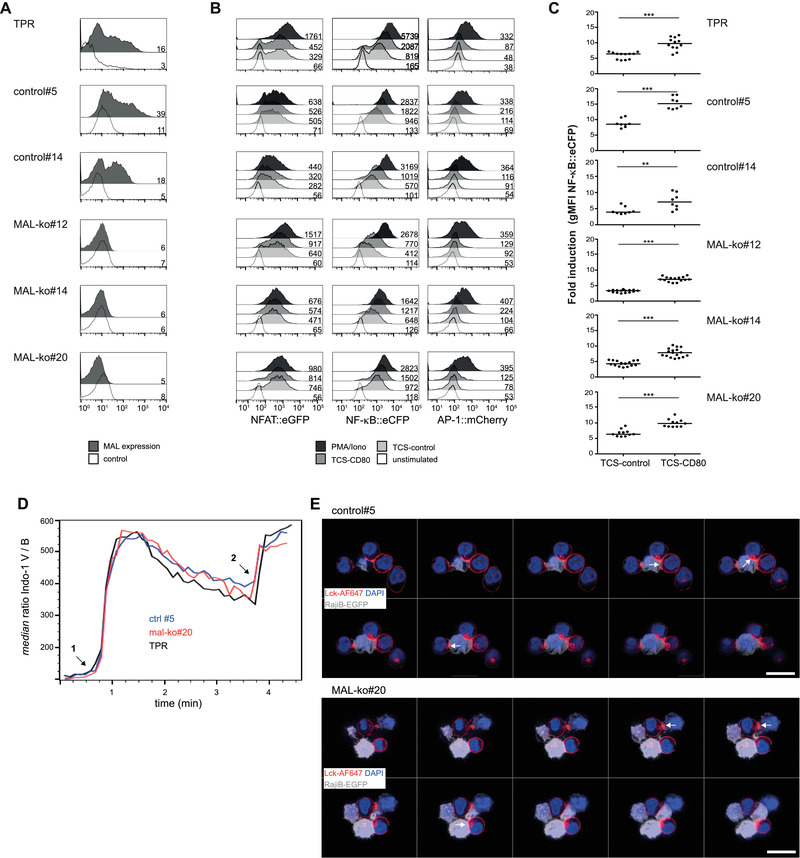
Impact of MAL ko on T‐cell activation. (A) Characterization of MAL ko Jurkat reporter cell lines by flow cytometry. MAL surface expression on parental Jurkat‐TPR clones, two CRISPR‐Cas9 treated control clones (control#5, #14) and three MAL‐CRISPR/Cas9‐ko clones (MAL‐ko#12, MAL‐ko#14, MAL‐ko#20) is shown. Open histogram: control staining (second antibody only), filled histogram: expression of MAL. (B) A representative experiment depicting reporter gene expression of parental TPR, control‐, and MAL‐ko clones. TPR were left either unstimulated or stimulated with TCS control, TCS expressing CD80 or PMA/ionomycin. NFAT::eGFP, NF‐κB::eCFP, and AP1::mCherry expression was measured via flow cytometry after 24 h. The gMFI value is shown for each histogram. (A, B) Data shown are representative for six independently performed experiments (n = 6). (C) TPR (as indicated) were stimulated with control TCS or TCS‐CD80. Reporter activation is shown as fold induction (gMFI of TCS stimulated cells/gMFI of unstimulated cells). Data from at least four independent experiments performed in duplicates and mean are shown (n≥4, ***p < 0.0001, **p < 0.001, two‐sided paired *t*‐test). (D) Indo‐1 labeled reporter cells were stimulated by (1) anti‐TCR‐beta chain mAb C305 followed by (2) ionomycin at 37°C and analyzed by flow cytometry. Shown are mean Indo‐1 violet/blue ratios of indicated cell lines. Data shown is representative for two independent experiments with triplicate measurements each. (E) Control and MAL‐ko clones were incubated with SEE‐coated Raji B cells expressing EGFP at 37°C to allow IS formation. T:APC pairs were adhered to fibronectin‐coated slides, fixated, permeabilized, and stained with anti‐Lck and AF647‐conjugated secondary antibody and DAPI. Afterwards confocal z‐stack analysis was performed. Ten consecutive stacks (≥0.5 μm apart) from representative synapses formed between control‐ and MAL‐ko clones and Raji cells are shown. Stacks with maximal Lck signals at the IS are marked with a white arrow. Data are representative for three independent experiments (n = 3). Scale bar, 20 μm.

To analyze the role of MAL in Lck transport and T‐cell signaling, we used a CRISPR‐Cas9 approach to knock out MAL in Jurkat T cells. To facilitate the functional evaluation of MAL deficiency, a Jurkat triple parameter reporter (TPR) platform developed by us and widely employed to assess T‐cell activation processes was used [22, 23, 24, 25]. This platform enables the simultaneous measurement of the activity of three transcription factors that play a major role in T‐cell activation, namely NF‐κB, NFAT, and AP‐1 via the expression levels of the fluorescent proteins eCFP, eGFP, and mCherry, respectively. Gene ko was confirmed by FACS analyses and Sanger sequencing (Fig. 8A and data not shown). MAL ko and control reporter cell lines were either left untreated or stimulated with T‐cell stimulator cells (TCS) expressing membrane‐bound CD3 single‐chain fragments with or without CD80, as described [26]. Additionally, all cell lines were activated with PMA/ionomycin. Reporter gene induction was analyzed by flow cytometry (Fig. 8B and C). The results of these experiments did not indicate a significant impairment of reporter gene induction in the MAL ko reporter cells compared to control reporter cells. We also investigated whether MAL ko cells exhibited alterations in TCR‐proximal signaling. Flow cytometric recording of Indo‐1 labeled control and ko cells revealed similar calcium flux responses after TCR cross‐linking in all clones analyzed, with no significant differences in response kinetics and magnitude between control and ko clones (representative fluxes and controls are shown in Fig. 8D, with the calcium flux experimental setup explained and statistics for all clones depicted in Supporting information Fig. S6A and B, respectively).

Because of the proposed function of MAL as transporter for Lck to the plasma membrane and consequently for its activity [12], we visualized by confocal laser‐scanning microscopy the effect of MAL on transport and localization of Lck in the TCR immune synapse (IS) by incubating parental, control‐clones and MAL ko reporter cells with SEE‐loaded Raji B‐cells. In line with our calcium flux results, we found no alterations in Lck plasma membrane localization and IS recruitment in MAL ko clones (representative images are shown in Fig. 8E, all other clones depicted in Supporting information Fig. S7).

Staining specificity control, IS enrichment score analysis and statistics of all clones analyzed are depicted in Supporting information Fig. S8.

Taken together our results do not support a basic role of MAL for instigation of TCR signaling and T‐cell activation in general, but may point toward an involvement of MAL in T cell differentiation.

## Discussion

By using the human TCR/CD3 complex as an immunogen in mice, we have generated mAb MT3, which discriminates subpopulations of human peripheral blood T lymphocytes: in particular, T‐cells producing IFN‐γ upon short‐term stimulation are MT3 surface‐negative, whereas IL‐2 producers mainly score positively. This interesting expression pattern prompted us to employ expression cloning to ascertain the MT3 antigen. Using this methodology, we identified the MT3 antigen as MAL, a highly hydrophobic integral membrane protein belonging to the family of proteolipid proteins. MAL is expressed in human T cells, myelin forming cells, polarized epithelial cells as well as in cancer cells and lymphomas [27, 28, 29]. Four different isoforms of MAL generated by alternative splicing were described, and we found that the mAb MT3 reacted exclusively with the large isoform A. We show that MAL‐A accounts for virtually 100% of the MAL gene transcripts in primary peripheral blood T lymphocytes and human T‐cell lines. Analysis of Jurkat T cells expressing a MAL‐eGFP fusion protein revealed that MAL was localized on the cell surface but also in intracellular compartments, which is in line with previous reports [12, 30].

Multiparameter surface expression profiling revealed that in human CD4^+^ T cells MAL was uniformly expressed on the naïve population. On the surface of memory cells, the majority of CCR7^+^ (CM) cells were MAL^+^, whereas most CCR7^–^ (EM) were found to be devoid of MAL surface expression. In comparison to the CD4^+^ subset, the CD8 subset contains much less MAL^+^ cells: in contrast to CD4^+^ T‐cells, naïve CD8^+^ T‐cells harbor a significant MAL negative population [10]. Thus, our results suggest a differential loss of MAL expression in the course of differentiation from the naïve to the EM type in human CD4^+^ and CD8^+^ T cells.

We performed a series of experiments to learn about the difference between MAL positive and MAL negative T cells. First, we separated MAL^+^ and MAL^–^ CD45RA^–^CD4^+^ memory T cells and performed qPCR analysis of transcription factors central for fate decision and T‐cell functions. These experiments revealed that the expression of TBX21, the gene that encodes T‐bet was much higher in MAL^–^ compared to MAL^+^ cells, whereas we did not obtain evidence for a differential regulation of any of the other transcription factors analyzed. T‐bet is the master regulator of Th1 cells and higher expression of IFN‐γ in MAL negative T cells would be consistent with a preferential expression of MAL in Th2/17 cells. To investigate this possibility, we performed seven color flow‐cytometric analysis to assess the expression of MAL in Th1 versus Th2 CD4^+^ T cells. Th2 cells did not contain higher percentages of MAL^+^ cells compared to Th1 cells. Instead, we observed that regardless of the T‐helper subset, the regulation of MAL was mainly determined by the differentiation state of CD4^+^ T‐cells: CM T cells were mainly MAL^+^, whereas EM T cells were mostly negative for MAL surface expression. Wambre and co‐workers recently identified Th2A cells, a population of CD4^+^ T‐cells, strongly associated with allergic disorders. These cells are terminally differentiated Th2 cells and concordant with our results these cells express lower levels of MAL compared to conventional Th2 cells [31]. Yu et al. demonstrated that nuclear mobilization of T‐bet was responsible for the rapid production of IFN‐γ in resting memory CD4^+^ T cells [32]. This data corroborate our findings that T‐bet positive EM T cells defined by loss of MAL are able to rapidly produce IFN‐γ.

Furthermore, we show that MAL expression is lost during activation of T cells, and CD3/CD28 stimulation experiments demonstrated that proliferating cells rapidly loose MAL surface expression. Collectively, our results suggest that surface expression of the MAL protein is associated with a nonactivated and nondifferentiated state. Naïve CD4^+^ T cells are uniformly MAL positive. Naïve CD8^+^ cells have a lower threshold of activation than naïve CD4^+^ cells [33, 34, 35] and, strikingly, only a fraction of CD8^+^CD45RA^+^ cells expresses MAL [10]. Thus, it is tempting to speculate that the latter subpopulation contains the real naïve CD8^+^ cells that transit via the MAL^–^CD45RA^+^ stage to the MAL^–^CD45RA^–^ memory type. Memory CD4^+^ T‐cells express less MAL than their naïve counterparts, hence, weaker signals suffice to activate these cells [36, 37]. Thus, MAL surface expression might correlate with a higher activation threshold in T cells.

The MAL protein is part of the apical sorting machinery and cycles between the trans‐Golgi network and the plasma membrane [38]. In human T cells, several functions have been attributed to MAL including protein sorting to the supramolecular activation cluster (SMAC) and regulation of exosome secretion [30, 39]. MAL was reported to be involved in the transport of Lck, the key signal‐generating tyrosine‐protein kinase of the TCR/CD3 complex, to the plasma membrane and to mediate its subcellular localization into lipid rafts [12]. Our phosphobiochemistry results indicate that MAL surface expression is negatively associated with Lck preactivation. MAL^–^ cells harbor comparable levels of pan‐Lck expression compared to MAL^+^ cells, however, in contrast to the MAL^+^ cells, they contain an enlarged pool of pS59‐pY394 double‐phosphorylated Lck, a hallmark signature of a TCR‐induced ERK‐feedback loop and obviously linked to IFN‐γ production and enhanced proliferation. Of note, MAL was reported to associate with microtubuli‐assisted transport of Lck to the plasma membrane in Jurkat cells and peripheral blood lymphocytes; synapse formation was impaired when MAL expression was more or less completely abolished by RNA knock‐down [12, 40]. However, as demonstrated by us here, peripheral blood CD4^+^ T cells not expressing the MT3 epitope on the cell surface represent highly active, more differentiated cells with robust proliferative capacity. Although it cannot be excluded that MT3 surface‐negative T cells possess intracellular MAL, mediating Lck transport, our data are difficult to reconcile with previous reports, which suggest an essential role of MAL in Lck transport and in TCR‐mediated signaling [12]. These claims were primarily based on results with JTIM cells, a Jurkat clone derived by chemical mutagenesis and lacking MAL expression, and on siRNA‐based knock‐down of MAL. We used CRISPR/Cas9 to knockout MAL‐A and all other isoforms by targeting exon 1 of MAL in a Jurkat reporter line for fluorescence monitoring the activation of NF‐κB, NFAT, and AP‐1, transcription factors that orchestrate T‐cell activation processes downstream of Lck. Reporter cell clones lacking MAL did not show a significant impairment in the activity of these transcription factors. Measurement of calcium flux responses indicated that TCR‐proximal signaling is also not compromised upon MAL deficiency. Importantly, membrane localization as well as IS recruitment of Lck was maintained in MAL ko reporter cells. Our results, which indicate that Lck can exert its essential function during T‐cell activation independent of MAL are in line with the outcome of genome‐wide CRISPR screens performed recently to identify essential regulators of human T‐cell stimulation responses. Screens in the Jurkat T‐cell line and in primary human T cells yielded high ranks for Lck (18 and 61) but not for MAL (10 690 and 7012) [41, 42]. The function of Lck is regulated at multiple levels and several molecules, including M6P/IGF2R, unc119, and Rab11‐FIP3, have been implicated in Lck localization [43, 44, 45, 46]. This redundancy might explain the maintained Lck transport in MAL knock out cells.

Although MAL was identified in human T cells more than 30 years ago, its expression in different T‐cell subsets was not studied to date. Of note, MAL expression is not detected in murine T cells (GSE122597_Gene_count_table), which supports our data that this molecule is dispensable for basic T‐cell activation processes. As we found that downregulation of MAL on human T cells is associated with higher proliferation and cytokine production that correlates with the sensitivity stages of the T cells, it tempts to speculate that MAL is not only phenotypically but also functionally involved in fine tuning the human T‐cell response.

## Material and methods

### Cells and cell culture

The study was approved by the Ethical Committees of the Faculty of Associated Medical Science, Chiang Mai University, Thailand (AMSSEC‐58EX‐090) and of the Medical University of Vienna (2001/2008). The study abides by the Declaration of Helsinki principles. PBMCs were isolated from buffy coats or heparinized blood obtained from healthy volunteer donors by using Ficoll–Hypaque (GE Healthcare Life Sciences, Pittsburgh, PA) density gradient centrifugation. PBMCs were cultured in RPMI1640 medium supplemented with 10% FBS (Thermo Fisher Scientific, Waltham, MA), 40 μg/mL gentamycin and 2.5 μg/mL amphotericin B in a humidified atmosphere of 5% CO_2_ at 37°C. The mouse thymoma cell line Bw5417 (short designation within this work Bw) and the human T‐cell lines HuT78; Jurkat E6.1 (Jkt E6.1), J.CaM1.6, HPB‐ALL, and MOLT‐4 were cultured as described [47]. TCS, which are based on the Bw cell line and stably express a CD3 single chain fragment have been described in detail [26]. Triple parameter reporter cell lines (TPR) are based on the Jkt E6.1 Jurkat cell line, stably expressing NF‐κB::eCFP, NFAT::eGFP, and AP‐1::mCherry reporter constructs that have been described previously [22].

### mAbs and flow cytometry

Generation of the mAb MT3 was described previously [10]. For binding studies, mAb MT3 was used at a final concentration of 20‐30 μg/mL and visualized by anti‐mouse IgM‐specific Abs (FITC/PE labeled from Beckman Coulter, Marseille, France; APC‐labeled from Jackson ImmunoResearch, West Grove, PA or Alexa Fluor 647‐labeled from Invitrogen, Eugene, OR). In some experiments DyLight‐649‐labelled anti‐mouse IgG‐H+L Abs (Jackson ImmunoResearch), which strongly bind to the light chains of IgM antibodies, were used for detection. CD3 mAb (OKT3) and CD28 mAb (Leu28) were purchased from Ortho Pharmaceuticals (Raritan, NJ) and BD Biosciences (San Jose, CA), respectively. Fluorochrome‐conjugated Abs used for polychromatic flow cytometry analysis were PerCP‐CD3 (UCHT1), PE/Cy7‐CD3 (UCHT1), PE/Dazzle 594‐CD4 (OKT4), Pacific Blue‐CD4 (RPA‐T4), Pacific Blue‐CD45RA mAb (HI100), BV‐711‐CD45RA mAb (HI100), Allophycocyanin‐anti‐CCR7 (G043H7), PE‐anti‐CCR4 (L291H4), PE/Cy7‐anti‐CXCR3 (G025H7), FITC‐CD45RA (HI100), PE‐anti‐IFN‐γ (B27), Alexa Fluor 488‐anti‐TNF‐α (Mab11), PE‐anti‐IL‐2 (MQ1‐17H12), PE‐anti‐IL‐4 (8D4‐8) (all purchased from BioLegend, San Diego, CA). PE‐CD45RO (UCHL1) mAb was obtained from BD Biosciences. PMA, ionomycin, and monensin were obtained from Sigma–Aldrich (St. Louis, MO). For intracellular cytokine staining, PBMCs were activated using 10 ng/mL PMA plus 1 μM ionomycin in the presence of 1.5 μM monensin for 6 h. Cells were washed once with staining buffer (PBS, 1% BSA, 0.02% NaN_3_). Subsequently, stimulated PBMCs were stained for CD3 and MT3 surface expression, washed and fixed with 4% paraformaldehyde‐PBS for 20 min at RT and washed twice with PBS and once with permeabilization buffer (PBS supplemented with 5% FBS, 0.1% saponin and 0.02% NaN_3_), and then incubated for 15 min at RT. Fixed cells were stained for IFN‐γ, IL‐2, IL‐4, or TNF‐α for 30 min at 4°C. Following a washing step with permeabilization buffer, cells were resuspended in staining buffer and subjected to flow analysis.

To exclude the TCS in the reporter assays, an APC‐labeled mCD45 (#104, Biolegend) antibody was used [48]. A DyLight‐649‐labelled goat‐anti‐mouse‐IgG (H+L) Ab (Jackson) was used to stain the anti‐CD3 single‐chain fragment on the TCS. Surface expression of CD80 on TCS was verified as described [26].

FACS analysis was done using a FACS Calibur, LSRFortessa (BD Biosciences) or a CyAn ADP flow cytometer (Beckman Coulter, Brea, CA) for seven‐color cytometry, according to recently published guidelines [49]. FlowJo software was used for flow cytometry data analysis (Tree Star, Inc. Ashland, OR).

### Identification of the MT3 antigen by expression cloning

Retroviral cDNA libraries generated from human DC and T cells were expressed in the Bw cell line as described previously [14, 15]. Transfected cell pools were stained with mAb MT3 followed by DyLight‐649‐labelled secondary Abs. Cells were then subjected for two rounds of sorting by a FACSAria cell sorter (BD Biosciences). Subsequently, single‐cell clones (SCCs) were established by limiting dilution culturing. Genomic DNA was prepared from MT3 reactive SCCs using Puregene (Gentra Systems, Minneapolis, MN) following the manufacturer's instructions. The retroviral cDNA inserts were PCR‐amplified from genomic DNA using primers specific for the flanking retroviral sequences as described [13]. PCR products were gel purified using the GeneJET Gel extraction kit (Fermentas, Burlington, ON, Canada). A PCR band of 0.7 kb obtained from several MT3‐reactive clones was cloned in a retroviral expression vector and re‐expressed in Bw cells using the retroviral expression plasmid pCJK2 as described [50]. The resultant cells were subsequently stained with mAb MT3 and analyzed by flow cytometry.

The retroviral cDNA insert was subjected to sequence analysis (Eurofins Genomics, Martinsried, Germany) and the obtained sequence was identified by BLAST analysis.

### Retroviral expression of MAL isoforms

The coding sequences of human MAL‐A, MAL‐B, MAL‐C, and MAL‐D were PCR‐amplified and cloned into a bicistronic retroviral expression vector as described [47]. Integrity of the constructs was confirmed by sequence analysis. Sequences identical for the coding region of the following Genbank entries were selected for further use: NM_002371 (MAL‐A), NM_022438 (MAL‐B), NM_022439 (MAL‐C), and NM_022440 (MAL‐D). These constructs were then stably expressed in the Bw cell line as described [50].

### Real time PCR analysis

Total RNA prepared from human T cells, flow‐sorted human T cells and human T‐cell lines using TRI^TM^‐Reagent was reversely transcribed using a first stand cDNA synthesis system (Fermentas) according to the manufacturers´ instructions. cDNA was amplified by using the following primer pairs for the MAL‐isoforms: MAL‐A: Mal‐a‐L (5’‐TGGGTGATGTTCGTGTCTGT‐3’) and Mal‐a‐R (5’‐TGAGGCGCTGAGGTAAAAGA‐3’), MAL‐B: Mal‐b‐L(5’‐CTGGGTCACCTTGGTGTTCT‐3’), and Mal‐b‐R (5’‐TTATGAAGACTTCCATCTGATTAAAGAG‐3’), MAL‐C: Mal‐c‐L (5’‐GCTCTTCATCTTTGAGTTTGACG‐3’) and Mal‐c‐R (5’‐TGAAGACTTCCATCTGATTAAAGAG‐3’), and MAL‐D: Mal‐d‐L (5’‐TGCTCTTCATCTTTGAGTTTGTG‐3’) and Mal‐d‐R (5’‐TTATGAAGACTTCCAT CTGATTAAAGAG‐3’). For real time‐PCR analysis of TBX21 (T‐bet; Th1) the following primer pairs were used: TBX21‐L (5’‐CCACCTGTTGTGGTCCAAGT) and TBX21‐R (5’‐AACATCCTGTAGTGGCTGGTG‐3’); cDNA (1 μL per reaction) was amplified using iQ SyBr‐Green Super Mix (Bio‐Rad Laboratories, Hercules, CA). Each primer was used at 5 μM. qPCR analyses were performed as described in detail [51]. For standardization of gene expression GAPDH was used. Normalized data are shown.

### Microscopy

A chimeric cDNA encoding MAL‐A fused to the C‐terminus of eGFP (EGFP‐MAL) was generated by overlap PCR and was cloned into the expression vector pCJK2. The construct was retrovirally expressed in Bw cells. Cells were stained for surface expression of MAL using mAb MT3 in conjunction with DyLight649‐labelled secondary Abs. Images were obtained using a DMI4000B microscope using LAS AF software (both from Leica Microsystems, Wetzlar, Germany) and ImageJ (NIH, Bethesda, MD).

For confocal z‐stack analysis of endogenous Lck in ISs in control‐ and MAL‐ko Jurkat clones, the cells were incubated with SEE‐loaded Raji B cells expressing lentivirally transduced pWPT‐GFP (Addgene #12255) in a 2:1 ratio for 15 min at 37°C. TPR clone:APC pairs were gently transferred onto plasma cleaned, fibronectin‐coated glass slides, and allowed to adhere before fixation (15 min in 8% PFA) and permeabilization (8 min in PBS 0.1% Triton X‐100). Upon washing, cells were incubated o/n at 4°C with 10 μg/mL anti‐Lck mAb 3A5 (SCBT, sc‐433) in PBS 3% BSA. Cells were washed trice and incubated for 1 h at room temperature with AF647‐labelled secondary Ab (1:400, goat antimouse IgG, LifeTechnologies, A21236) and counterstained with DAPI. Confocal z‐stack analysis was performed with a LSM780 apparatus (Zeiss) employing a 63x/1.4 Plan‐Apochromat/Oil/DIC objective and operated with the ZEN2.3 SP1 software. Approximately 20 individual synapses per clone:APC pair were imaged for Lck IS enrichment score as outlined in Supporting information Fig. S7.

### CFSE‐based T‐cell proliferation assay

CD4^+^CD45RO^+^MT3^+^ and CD4^+^CD45RO^–^MT3^+^ cells were isolated from PBMCs using an SH800S Cell sorter (Sony Biotechnology, Japan) with a purity of >98%. T cells (1 × 10^7^ cells/mL) were CFSE‐labeled as described previously [50]. CFSE‐labeled T cells (5 × 10^5^ cells/mL) were stimulated with immobilized CD3 mAb OKT3 (1 μg/mL) and CD28 mAb Leu28 (2 μg/mL). Following 3, 6, and 9 days of stimulation, coexpression of MT3 and CD45RO was analyzed. Cells were analyzed on a BD Flow Cytometer (BD Biosciences).

### Phosphobiochemistry

MAL^–^ and MAL^+^ CD4^+^ T cells were sorted from surface‐stained PBMCs as indicated in Supporting information Fig. S5 and rested overnight RPMI 1640 medium supplemented with 5% FCS. Ninety min before stimulation, cells were transferred to RPMI 1640 medium supplemented with 1% FCS. Cells were surface‐labeled with 10 μg/mL OKT3 on ice and stimulated by addition of 5 μg/mL goat antimouse IgG + M F(ab')_2_ (Jackson ImmunoResearch) and incubation at 37°C for 3 min or left unstimulated. Total cell lysates were generated and subjected to reducing SDS‐PAGE (10%) and Western blot analysis as described [52] with addition of 20 μM PP2 Src kinase inhibitor (Sigma‐Aldrich) to avoid artefactual phosphorylation during cell lysis [53]. PageRuler Prestained Protein Ladder (Thermo Scientific) was used as size marker. Membranes were probed with pY416‐Src Ab (#2101) recognizing the pY394‐Lck activatory epitope followed by chemiluminescent detection of HRP‐linked anti‐rabbit IgG (#7074, all Cell Signaling Technology, Daners, MA) via the LAS 4000 (Fujifilm, Tokyo, Japan) device. Upon stripping, washing and reblocking, membranes were reprobed with anti‐Lck mAb 3A5 (Santa Cruz Biotechnology, Dallas, TX) and anti‐mouse IgG‐Peroxidase Abs (A9044, Sigma–Aldrich) to visualize pan‐Lck expression. Densitometric analysis was performed using the ImageJ (NIH) software.

### CRISPR‐Cas9 approach targeting MAL‐A

crRNA targeting MAL‐A (NM_002371, 3’‐GTGAAGACCGAGAAGCCACT‐5’ Hs.Cas9.MAL.1.AD), control crRNA (negative control crRNA#1, #1072544), Alt‐R S.p. HiFi Cas9 Nuclease 3NLS and tracrRNA were purchased from IDT (Integrated DNA Technologies Coralville, IA). cr:trcrRNA duplex formation and Cas9/RNP assembly was done as described [54]. Jurkat E6.1‐TPR cells were electroporated using the Amaxa^®^Nucleofector^®^ system (Lonza, Basel, Switzerland) program S‐018. Single cell clones (SCCs) (MAL‐A targeted and controls) were established via limiting dilution. PCR inserts of selected clones were retrieved from genomic DNA (forward: 5’‐GAAGAGGTTCAGGGCGGTGC‐3’, reverse: CCCCTCATTCTGTTGGTCTAGAAG). To confirm gene ko, Sanger sequencing (using the same primers as described above) and flow cytometry were performed. Sequencing data were analyzed using TIDEdeskgen webtool [55].

### Reporter assay

Jurkat‐TPR MAL ko and control cell lines (5 × 10^4^) were stimulated with PMA (100 ng/mL) and ionomycin (1 μM) or with TCS (TCS‐control or TCS‐CD80; 2 × 10^4^). Following 24 h of coculture, reporter activity was analyzed by FACS as described previously [22]. Normalized data show the ratio of the eCFP‐geometric mean fluorescence intensity (gMFI) values of the stimulated and unstimulated conditions.

### Calcium flux

To enable simultaneous measurement of control‐ and MAL‐ko TPR clones and exclude handling‐induced variations with regard to Indo labeling and stimulation, cells were labeled separately either with DMSO (“mock”), CFSE or eF670 using a standard protocol and rested overnight in RPMI 1640 medium supplemented with 5% FCS. Upon washing, these cells, each stained with one of the three different labels were mixed per sample and labeled with 2 μM Indo‐1 AM in 150 μL standard RPMI 1640 medium per 1×10^6^ cells for 30 min at 37°C. Cells were washed and rested at 1.5 × 10^6^/mL for another 30 min at 37°C. Afterwards, 500 μL aliquots were kept on ice for 10 min and preheated for 5 min at 37°C before measurement. Indo‐1 violet (Ca^2+^ bound, UV‐1 fluorescence channel) and blue (no Ca^2+^ bound, UV‐7 fluorescence channel) emission was recorded by a Cytek Aurora flow cytometer (Cytek Biosciences, Fremont, CA) at 37°C for 45 s. IgM mAb C305 recognizing the Jurkat TCRβ epitope (a kind gift from Art Weiss) was added (1:600 final dilution) and recording was continued for 4 min before 4 μM ionomycin was added. Recording was stopped 45 s after addition of the ionophore positive control. From all recorded singlet core events, control and ko populations were gated according to CFSE and eF670 (double‐negative and single‐positive) signals as outlined in Supporting information Fig. 6A using the FlowJo v10.6.2 software. The mean Indo‐violet/blue (V/B) ratio was plotted versus time to depict calcium flux kinetics of control and ko cells during stimulation. To enable comparison between individual samples and experiments, mean Indo‐V/B ratio during the maximum peak upon mAb C305 addition was divided by the ratio recorded during baseline period. Per clone, mean fold‐induction post‐mAbC305 stimulation ± SD was calculated from three independent experiments.

### Statistics

Using GraphPad Prism (Version 5, GraphPad Software, Inc., La Jolla, CA), data were analyzed by a two‐sided paired *t*‐test or by one‐way ANOVA followed by a Tukey's posthoc test.

## Authorship

K.M. and J.L. performed experiments shown in Fig. 1–6, 8. P.S. performed phospho‐biochemical, calcium flux, and microscopy experiments (Figs. 7 and 8). K.M., J.L., P.S. K.P., S.P., and P.St. analyzed data. P.St. and H.S. supervised experimental work. P.St., H.S., W.K., S.P., and V.L. assisted with experimental design and provided reagents. All authors wrote and critically revised the manuscript.

### Peer review

The peer review history for this article is available at https://publons.com/publon/10.1002/eji.202048603.

## Conflict of statement

The authors declare no financial or commercial conflict of interest.

**Abbreviations**
CFSE: carboxyfluorescein succinimidyl ester
CM: central memory
EM: effector memory
IS: immunological synapse
Lck: lymphocyte‐specific kinase
MAL: myelin‐and‐lymphocyte protein
TPR: triple parameter reporter

## Supporting information

Supporting Information

## Data Availability

The data that support the findings of this study are available from the corresponding authors upon reasonable request.

## References

[1] Bunnell, S. C. , Hong, D. I. , Kardon, J. R. , Yamazaki, T. , McGlade, C. J. , Barr, V. A. and Samelson, L. E. , T cell receptor ligation induces the formation of dynamically regulated signaling assemblies. J. Cell Biol. 2002. 158: 1263–1275. 12356870 10.1083/jcb.200203043 PMC2173229

[2] Weiss, A. , Shields, R. , Newton, M. , Manger, B. and Imboden, J. , Ligand‐receptor interactions required for commitment to the activation of the interleukin 2 gene. J. Immunol. 1987. 138: 2169–2176. 3104454

[3] Crabtree, G. R. , Contingent genetic regulatory events in T lymphocyte activation. Science 1989. 243: 355–361. 2783497 10.1126/science.2783497

[4] Samelson, L. E. , Patel, M. D. , Weissman, A. M. , Harford, J. B. and Klausner, R. D. , Antigen activation of murine T cells induces tyrosine phosphorylation of a polypeptide associated with the T cell antigen receptor. Cell 1986. 46: 1083–1090. 2428504 10.1016/0092-8674(86)90708-7

[5] Dustin, M. L. , Olszowy, M. W. , Holdorf, A. D. , Li, J. , Bromley, S. , Desai, N. , Widder, P. et al., A novel adaptor protein orchestrates receptor patterning and cytoskeletal polarity in T‐cell contacts. Cell 1998. 94: 667–677. 9741631 10.1016/s0092-8674(00)81608-6

[6] Monks, C. R. , Freiberg, B. A. , Kupfer, H. , Sciaky, N. and Kupfer, A. , Three‐dimensional segregation of supramolecular activation clusters in T cells. Nature 1998. 395: 82–86. 9738502 10.1038/25764

[7] Grakoui, A. , Bromley, S. K. , Sumen, C. , Davis, M. M. , Shaw, A. S. , Allen, P. M. and Dustin, M. L. , The immunological synapse: a molecular machine controlling T cell activation. Science 1999. 285: 221–227. 10398592 10.1126/science.285.5425.221

[8] Sprent, J. and Surh, C. D. , T cell memory. Annu. Rev. Immunol. 2002. 20: 551–579. 11861612 10.1146/annurev.immunol.20.100101.151926

[9] Farber, D. L. , Biochemical signaling pathways for memory T cell recall. Semin. Immunol. 2009. 21: 84–91. 19298946 10.1016/j.smim.2009.02.003 PMC2683752

[10] Mahasongkram, K. , Pata, S. , Chruewkamlow, N. and Kasinrerk, W. , Identification of a T cell surface molecule using a monoclonal antibody produced by TCR/CD3 complex immunization. Asian Pac J Allergy Immunol 2015. 33: 107–116. 26141031 10.12932/AP0538.33.2.2015

[11] Sanchez‐Pulido, L. , Martin‐Belmonte, F. , Valencia, A. and Alonso, M. A. , MARVEL: a conserved domain involved in membrane apposition events. Trends Biochem. Sci. 2002. 27: 599–601. 12468223 10.1016/s0968-0004(02)02229-6

[12] Anton, O. , Batista, A. , Millan, J. , Andres‐Delgado, L. , Puertollano, R. , Correas, I. and Alonso, M. A. , An essential role for the MAL protein in targeting Lck to the plasma membrane of human T lymphocytes. J. Exp. Med. 2008. 205: 3201–3213. 19064697 10.1084/jem.20080552 PMC2605221

[13] Steinberger, P. , Majdic, O. , Derdak, S. V. , Pfistershammer, K. , Kirchberger, S. , Klauser, C. , Zlabinger, G. et al., Molecular characterization of human 4Ig‐B7‐H3, a member of the B7 family with four Ig‐like domains. J. Immunol. 2004. 172: 2352–2359. 14764704 10.4049/jimmunol.172.4.2352

[14] Pfistershammer, K. , Lawitschka, A. , Klauser, C. , Leitner, J. , Weigl, R. , Heemskerk, M. H. , Pickl, W. F. et al., Allogeneic disparities in immunoglobulin‐like transcript 5 induce potent antibody responses in hematopoietic stem cell transplant recipients. Blood 2009. 114: 2323–2332. 19617579 10.1182/blood-2008-10-183814

[15] Popow, I. , Leitner, J. , Grabmeier‐Pfistershammer, K. , Majdic, O. , Zlabinger, G. J. , Kundi, M. and Steinberger, P. , A comprehensive and quantitative analysis of the major specificities in rabbit antithymocyte globulin preparations. Am. J. Transplant. 2013. 13: 3103–3113. 24168235 10.1111/ajt.12514

[16] Alonso, M. A. and Weissman, S. M. , cDNA cloning and sequence of MAL, a hydrophobic protein associated with human T‐cell differentiation. Proc Natl Acad Sci USA 1987. 84: 1997–2001. 3494249 10.1073/pnas.84.7.1997 PMC304570

[17] Rancano, C. , Rubio, T. and Alonso, M. A. , Alternative splicing of human T‐cell‐specific MAL mRNA and its correlation with the exon/intron organization of the gene. Genomics 1994. 21: 447–450. 8088843 10.1006/geno.1994.1294

[18] Salmond, R. J. , Filby, A. , Qureshi, I. , Caserta, S. and Zamoyska, R. , T‐cell receptor proximal signaling via the Src‐family kinases, Lck and Fyn, influences T‐cell activation, differentiation, and tolerance. Immunol Rev. 2009. 228: 9–22. 19290918 10.1111/j.1600-065X.2008.00745.x

[19] Stefanova, I. , Hemmer, B. , Vergelli, M. , Martin, R. , Biddison, W. E. and Germain, R. N. , TCR ligand discrimination is enforced by competing ERK positive and SHP‐1 negative feedback pathways. Nat. Immunol. 2003. 4: 248–254. 12577055 10.1038/ni895

[20] Poltorak, M. , Arndt, B. , Kowtharapu, B. S. , Reddycherla, A. V. , Witte, V. , Lindquist, J. A. , Schraven, B. et al., TCR activation kinetics and feedback regulation in primary human T cells. Cell Commun. Signaling 2013. 11 10.1186/1478-811X-11-4 PMC3842781 23317458

[21] Dutta, D. , Barr, V. A. , Akpan, I. , Mittelstadt, P. R. , Singha, L. I. , Samelson, L. E. and Ashwell, J. D. , Recruitment of calcineurin to the TCR positively regulates T cell activation. Nat. Immunol. 2017. 18: 196–204. 27941787 10.1038/ni.3640 PMC6352896

[22] Jutz, S. , Leitner, J. , Schmetterer, K. , Doel‐Perez, I. , Majdic, O. , Grabmeier‐Pfistershammer, K. , Paster, W. et al., Assessment of costimulation and coinhibition in a triple parameter T cell reporter line: simultaneous measurement of NF‐kappaB, NFAT and AP‐1. J. Immunol. Methods 2016. 430: 10–20. 26780292 10.1016/j.jim.2016.01.007

[23] Rydzek, J. , Nerreter, T. , Peng, H. , Jutz, S. , Leitner, J. , Steinberger, P. , Einsele, H. et al., Chimeric antigen receptor library screening using a novel NF‐kappaB/NFAT reporter cell platform. Mol. Ther. 2019. 27: 287–299. 30573301 10.1016/j.ymthe.2018.11.015 PMC6369451

[24] Abdelaziz, M. O. , Ossmann, S. , Kaufmann, A. M. , Leitner, J. , Steinberger, P. , Willimsky, G. , Raftery, M. J. et al., Development of a human cytomegalovirus (HCMV)‐based therapeutic cancer vaccine uncovers a previously unsuspected viral block of MHC class i antigen presentation. Front Immunol 2019. 10: 1776 31417555 10.3389/fimmu.2019.01776 PMC6682651

[25] Dufva, O. , Koski, J. , Maliniemi, P. , Ianevski, A. , Klievink, J. , Leitner, J. , Polonen, P. et al., Integrated drug profiling and CRISPR screening identify essential pathways for CAR T cell cytotoxicity. Blood 2019. 135: 597‐609. 10.1182/blood.2019002121 PMC7098811 31830245

[26] Leitner, J. , Kuschei, W. , Grabmeier‐Pfistershammer, K. , Woitek, R. , Kriehuber, E. , Majdic, O. , Zlabinger, G. et al., T cell stimulator cells, an efficient and versatile cellular system to assess the role of costimulatory ligands in the activation of human T cells. J. Immunol. Methods 2010. 362: 131–141. 20858499 10.1016/j.jim.2010.09.020 PMC2975062

[27] Perez, P. , Puertollano, R. and Alonso, M. A . Structural and biochemical similarities reveal a family of proteins related to the MAL proteolipid, a component of detergent‐insoluble membrane microdomains. Biochem. Biophys. Res. Commun. 1997. 232: 618–621. 9126323 10.1006/bbrc.1997.6338

[28] Horne, H. N. , Lee, P. S. , Murphy, S. K. , Alonso, M. A. , Olson, J. A., Jr. and Marks, J. R . Inactivation of the MAL gene inbreast cancer is a common event that predicts benefit from adjuvant chemotherapy. Mol. Cancer Res. 2009. 7: 199–209. 19208741 10.1158/1541-7786.MCR-08-0314 PMC2700346

[29] Copie‐Bergman, C. , Plonquet, A. , Alonso, M. A. , Boulland, M. L. , Marquet, J. , Divine, M. , Moller, P. et al., MAL expression in lymphoid cells: further evidence for MAL as a distinct molecular marker of primary mediastinal large B‐cell lymphomas. Mod Pathol. 2002. 15: 1172–1180. 12429796 10.1097/01.MP.0000032534.81894.B3

[30] Anton, O. M. , Andres‐Delgado, L. , Reglero‐Real, N. , Batista, A. and Alonso, M. A. , MAL protein controls protein sorting at the supramolecular activation cluster of human T lymphocytes. J. Immunol. 2011. 186: 6345–6356. 21508261 10.4049/jimmunol.1003771

[31] Wambre, E. , Bajzik, V. , DeLong, J. H. , O'Brien, K. , Nguyen, Q. A. , Speake, C. , Gersuk, V. H. et al., A phenotypically and functionally distinct human TH2 cell subpopulation is associated with allergic disorders. Sci. Transl. Med. 2017. 9. 10.1126/scitranslmed.aam9171 PMC5987220 28768806

[32] Yu, S. F. , Zhang, Y. N. , Yang, B. Y. and Wu, C. Y. , Human memory, but not naive, CD4+ T cells expressing transcription factor T‐bet might drive rapid cytokine production. J. Biol. Chem. 2014. 289: 35561–35569. 25378399 10.1074/jbc.M114.608745 PMC4271239

[33] Obst, R. , van Santen, H. M. , Mathis, D. and Benoist, C. , Antigen persistence is required throughout the expansion phase of a CD4(+) T cell response. J. Exp. Med. 2005. 201: 1555–1565. 15897273 10.1084/jem.20042521 PMC2212918

[34] Williams, M. A. and Bevan, M. J. , Shortening the infectious period does not alter expansion of CD8 T cells but diminishes their capacity to differentiate into memory cells. J. Immunol. 2004. 173: 6694–6702. 15557161 10.4049/jimmunol.173.11.6694

[35] Kaech, S. M. , Wherry, E. J. and Ahmed, R. , Effector and memory T‐cell differentiation: implications for vaccine development. Nat. Rev. Immunol. 2002. 2: 251–262. 12001996 10.1038/nri778

[36] Zehn, D. , King, C. , Bevan, M. J. and Palmer, E. , TCR signaling requirements for activating T cells and for generating memory. Cell. Mol. Life Sci. 2012. 69: 1565–1575. 22527712 10.1007/s00018-012-0965-x PMC11114768

[37] Chandok, M. R. and Farber, D. L. , Signaling control of memory T cell generation and function. Semin. Immunol. 2004. 16: 285–293. 15528073 10.1016/j.smim.2004.08.009

[38] Puertollano, R. and Alonso, M. A. , MAL, an integral element of the apical sorting machinery, is an itinerant protein that cycles between the trans‐Golgi network and the plasma membrane. Mol. Biol. Cell 1999. 10: 3435–3447. 10512878 10.1091/mbc.10.10.3435 PMC25613

[39] Ventimiglia, L. N. , Fernandez‐Martin, L. , Martinez‐Alonso, E. , Anton, O. M. , Guerra, M. , Martinez‐Menarguez, J. A. , Andres, G. et al., Cutting edge: regulation of exosome secretion by the integral MAL protein in T cells. J. Immunol. 2015. 195: 810–814. 26109641 10.4049/jimmunol.1500891

[40] Andres‐Delgado L , Anton, O. M. , Madrid, R. , Byrne, J. A. and Alonso, M. A . Formin INF2 regulates MAL‐mediated transport of Lck to the plasma membrane of human T lymphocytes. Blood 2010. 116: 5919–5929. 20881207 10.1182/blood-2010-08-300665

[41] Shifrut, E. , Carnevale, J. , Tobin, V. , Roth, T. L. , Woo, J. M. , Bui, C. T. , Li, P. J. et al., Genome‐wide CRISPR screens in primary human T cells reveal key regulators of immune function. Cell 2018. 175: 1958–1971 e1915 30449619 10.1016/j.cell.2018.10.024 PMC6689405

[42] Shang, W. , Jiang, Y. , Boettcher, M. , Ding, K. , Mollenauer, M. , Liu, Z. , Wen, X. et al., Genome‐wide CRISPR screen identifies FAM49B as a key regulator of actin dynamics and T cell activation. Proc Natl Acad Sci USA 2018. 115: E4051–E4060. 29632189 10.1073/pnas.1801340115 PMC5924929

[43] Gorska, M. M. , Stafford, S. J. , Cen, O. , Sur, S. and Alam, R. , Unc119, a novel activator of Lck/Fyn, is essential for T cell activation. J. Exp. Med. 2004. 199: 369–379. 14757743 10.1084/jem.20030589 PMC2211793

[44] Pfisterer, K. , Forster, F. , Paster, W. , Supper, V. , Ohradanova‐Repic, A. , Eckerstorfer, P. , Zwirzitz, A. et al., The late endosomal transporter CD222 directs the spatial distribution and activity of Lck. J. Immunol. 2014. 193: 2718–2732. 25127865 10.4049/jimmunol.1303349

[45] Simeoni, L. , Lck activation: puzzling the pieces together. Oncotarget 2017. 8: 102761–102762. 29262519 10.18632/oncotarget.22309 PMC5732685

[46] Bouchet, J. , Del Rio‐Iniguez, I. , Vazquez‐Chavez, E. , Lasserre, R. , Aguera‐Gonzalez, S. , Cuche, C. , McCaffrey, M. W. et al., Rab11‐FIP3 regulation of Lck endosomal traffic controls TCR signal transduction. J. Immunol. 2017. 198: 2967–2978. 28235866 10.4049/jimmunol.1600671

[47] Leitner, J. , Reutner, K. , Essler, S. E. , Popow, I. , Gerner, W. , Steinberger, P. and Saalmuller, A. , Porcine SWC1 is CD52–final determination by the use of a retroviral cDNA expression library. Vet Immunol Immunopathol. 2012. 146: 27–34. 22336037 10.1016/j.vetimm.2012.01.012 PMC3334673

[48] De Sousa Linhares, A. , Battin, C. , Jutz, S. , Leitner, J. , Hafner, C. , Tobias, J. , Wiedermann, U. et al., Therapeutic PD‐L1 antibodies are more effective than PD‐1 antibodies in blocking PD‐1/PD‐L1 signaling. Sci. Rep. 2019. 9: 11472 31391510 10.1038/s41598-019-47910-1 PMC6685986

[49] Cossarizza, A. , Chang, H. D. , Radbruch, A. , Acs, A. , Adam, D. , Adam‐Klages, S. , Agace, W. W. et al., Guidelines for the use of flow cytometry and cell sorting in immunological studies (second edition). Eur. J. Immunol. 2019. 49: 1457–1973. 31633216 10.1002/eji.201970107 PMC7350392

[50] Kober, J. , Leitner, J. , Klauser, C. , Woitek, R. , Majdic, O. , Stockl, J. , Herndler‐Brandstetter, D. et al., The capacity of the TNF family members 4‐1BBL, OX40L, CD70, GITRL, CD30L and LIGHT to costimulate human T cells. Eur. J. Immunol. 2008. 38: 2678–2688. 18825741 10.1002/eji.200838250 PMC2975061

[51] Leitner, J. , Grabmeier‐Pfistershammer, K. , Majdic, O. , Zlabinger, G. and Steinberger, P. , Interaction of antithymocyte globulins with dendritic cell antigens. Am. J. Transplant. 2011. 11: 138–145. 21199353 10.1111/j.1600-6143.2010.03322.x

[52] Paster, W. , Paar, C. , Eckerstorfer, P. , Jakober, A. , Drbal, K. , Schutz, G. J. , Sonnleitner, A. et al., Genetically encoded Forster resonance energy transfer sensors for the conformation of the Src family kinase Lck. J. Immunol. 2009. 182: 2160–2167. 19201869 10.4049/jimmunol.0802639

[53] Ballek, O. , Valecka, J. , Manning, J. and Filipp, D . The pool of preactivated Lck in the initiation of T‐cell signaling: a critical re‐evaluation of the Lck standby model. Immunol Cell Biol 2015. 93: 384–395. 25420722 10.1038/icb.2014.100

[54] Seki, A. and Rutz, S. , Optimized RNP transfection for highly efficient CRISPR/Cas9‐mediated gene knockout in primary T cells. J. Exp. Med. 2018. 215: 985–997. 29436394 10.1084/jem.20171626 PMC5839763

[55] Brinkman, E. K. , Chen, T. , Amendola, M. and van Steensel, B. , Easy quantitative assessment of genome editing by sequence trace decomposition. Nucleic Acids Res. 2014. 42: e168 25300484 10.1093/nar/gku936 PMC4267669

