# Supplementary material for: Differentiation and activation of human CD4 T cells is associated with a gradual loss of myelin and lymphocyte protein
Source: Eur J Immunol. 2021 Jan 25;51(4):848–63. doi: 10.1002/eji.202048603 (PMC8248321; doi:10.1002/eji.202048603)
Supplement: Supplementary file 1 — Supporting Information [file EJI-51-848-s001.docx]

**Supplementary Information**

**Differentiation and activation of human CD4 T-cells is associated with a gradual loss of Myelin and Lymphocyte Protein**

Judith Leitner^1#^, Kodchakorn Mahasongkram^2#^, Philipp Schatzlmaier^3#^, Karin Pfisterer^3,4^, Vladimir Leksa^3,5^, Supansa Pata^2,6^, Watchara Kasinrerk^2,6^, Hannes Stockinger^3^, Peter Steinberger^1^

^1^Division of Immune Receptors and T Cell Activation, Institute of Immunology, Center for Pathophysiology, Infectiology and Immunology, Medical University of Vienna, Lazarettgasse 19, 1090 Vienna, Austria,

^2^Division of Clinical Immunology, Department of Medical Technology, Faculty of Associated Medical Sciences, Chiang Mai University, Chiang Mai, Thailand

^3^Institute for Hygiene and Applied Immunology, Centre for Pathophysiology, Infectiology and Immunology, Medical University of Vienna, Lazarettgasse 19, 1090 Vienna, Austria

^4^current: Department of Dermatology, Medical University of Vienna, Vienna, 1090, Austria

^5^Laboratory of Molecular Immunology, Institute of Molecular Biology, Slovak Academy of Sciences, Bratislava, Slovakia

^6^Biomedical Technology Research Centre, National Centre for Genetic Engineering and Biotechnology, National Science and Technology Development Agency at the Faculty of Associated Medical Sciences, Chiang Mai University, Chiang Mai, Thailand

# these authors contributed equally to this work

correspondence to Peter Steinberger ([peter.steinberger@meduniwien.ac.at](mailto:peter.steinberger@meduniwien.ac.at)) or Hannes Stockinger ([hannes.stockinger@meduniwien.ac.at](mailto:hannes.stockinger@meduniwien.ac.at)).


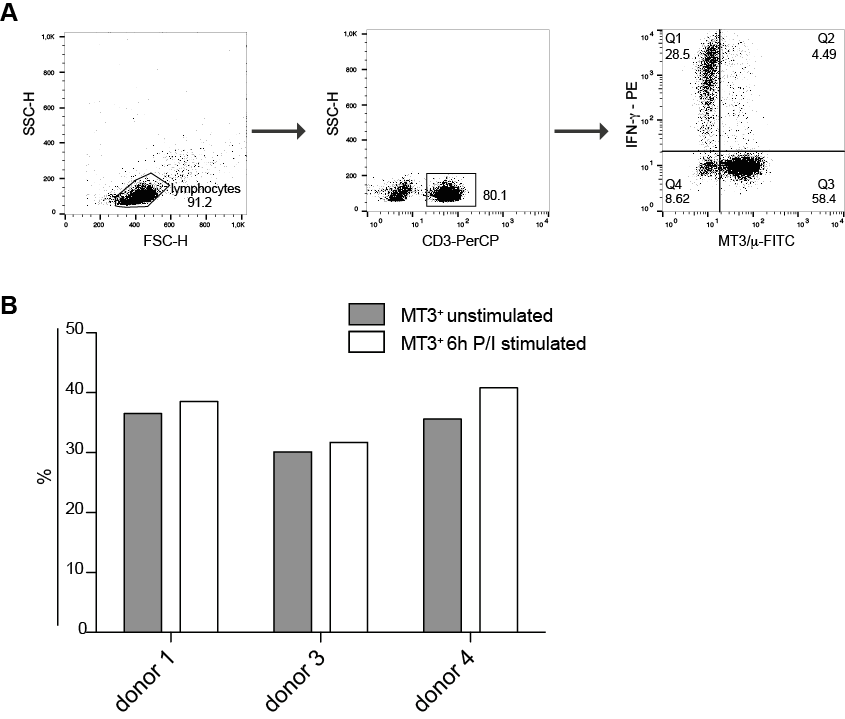


**Suppl. Fig. 1.**

A) Gating strategy to Fig. 1A is shown. Viable cells were identified by their forward/side scatter properties. Then, CD3^+^ cells were gated and analysed for co-expression of MT3 and cytokines.

B) Percentage of MT3 expression in unstimulated versus stimulated (6h PMA/Ionomycin – P/I) human T cells is shown.

**
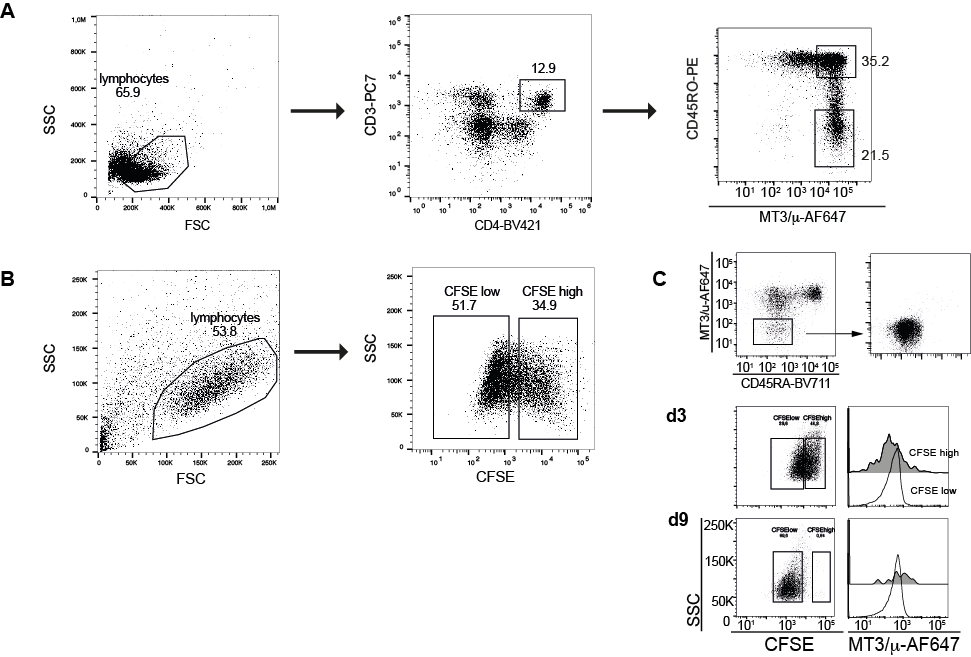
**

**Suppl. Fig. 2.**

A) Gating strategy to Fig. 4A is shown. Viable cells were identified by their forward/side scatter properties. Then MT3^+^ CD3^+^CD4^+^CD45RO^+^ memory cells) and CD3^+^CD4^+^CD45RO^-^ (naive cells) were isolated by flow sorting.

B) Gating strategy to Fig. 4B is shown. Isolated cells (shown in A) were CFSE-labelled and stimulated with immobilized CD3 and CD28 mAbs. At the indicated time points cells were analysed by flow cytometry. Viable cells were identified by their forward/side scatter properties. Next, the expression of CD45RO and MAL within the CFSE high and low subset was analysed.

C) The MT3^-^CD45RA^-^CD3^+^ human T-cell subset was sorted, labelled with CFSE and stimulated with immobilized CD3 and CD28 mAbs. At the indicated time points cells were harvested, stained for CD45RO and MAL surface expression and analysed by flow cytometry. Cells were gated on CFSE^high^ (filled histogram) and CFSE^low^ (open histogram) and analysed for their MAL expression. Data of one donor representative of five analysed are shown.


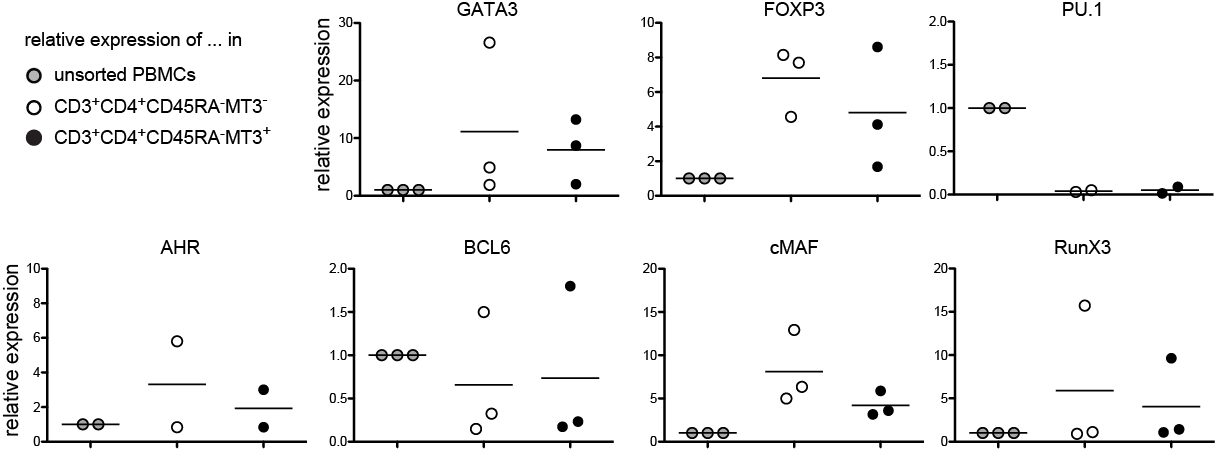


**Suppl. Fig. 3.**

Expression of GATA3, FOXP3, PU.1, AHR, BCL6, cMAF and RunX3 in unsorted

PBMCs and in the MT3^-^- and MT3^+^ -CD4^+^CD45RA^-^ T cells was analysed by qPCR. Data shown represent mean of two (for PU.1, AHR) or three donors (all other transcription factors analysed).


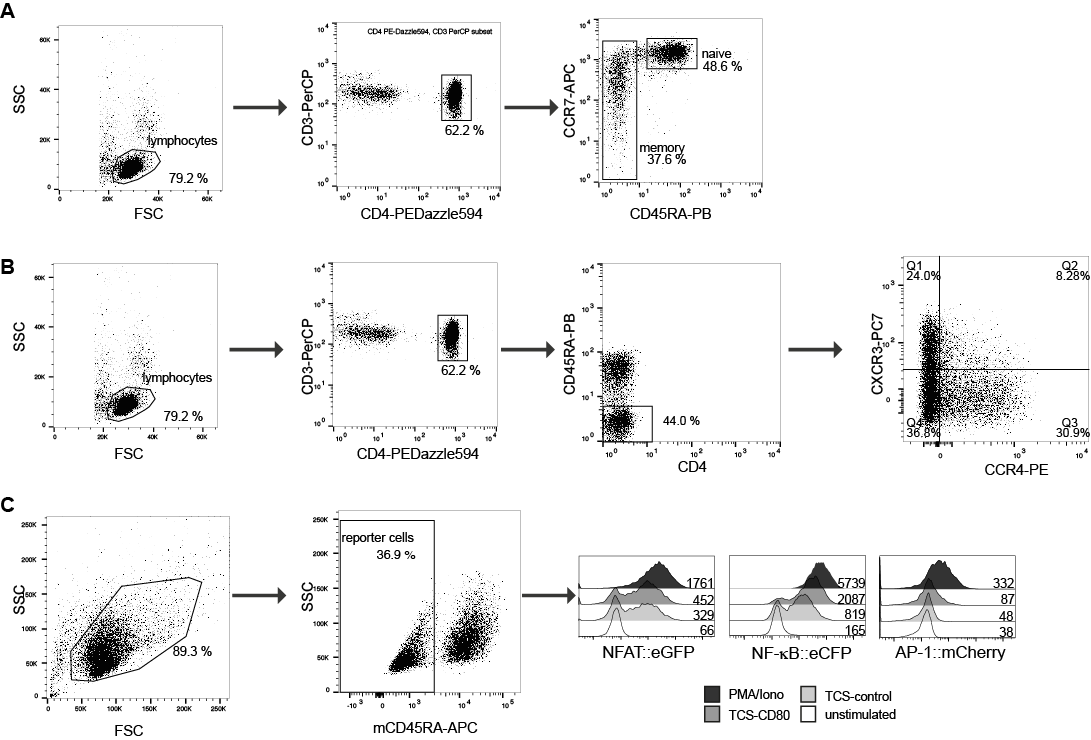


**Suppl. Fig.4.**

A,B) Human PBMCs were co-stained with mAbs to CD3, CD4, CD45RA, CCR7, CXCR3, CCR4 and MT3. Gating strategy to Figure 6A (A) Figure 6C (B) and is shown.

C) Gating strategy for the reporter assay shown in Figure 8B. Viable cells were identified by their forward/side scatter properties. Next, stimulator cells were excluded by staining for mCD45RA expression. eGFP, eCFP and mCherry expression of the reporter cells was analysed by flow cytometry.

**
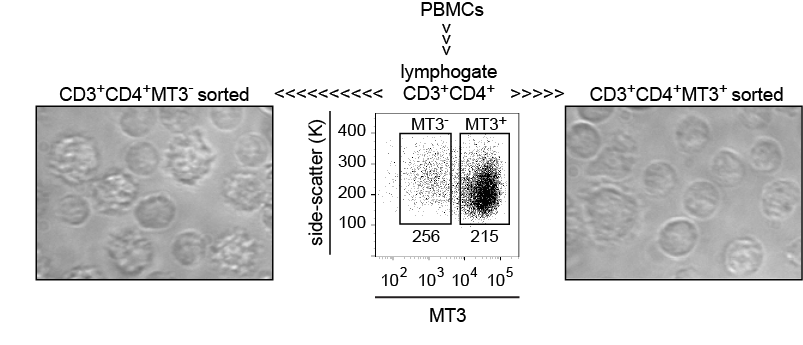
Suppl. Fig.5.**

Gating strategy and bright-field microscopy of MAL^+^ versus MAL^-^ CD3^+^CD4^+^T cells. In line with their CD45R expression profile and reduced proliferative response, the majority of MT3^+^ sorted CD3^+^CD4^+^ T cells appears as small, round-shaped “naïve” cells under the light microscope (right panel). The MT3^-^ CD3^+^CD4^+^ T cells (left panel) feature less symmetric and more protruded membranes in agreement with their enhanced side-scatter signals in flow cytometry. Mean of side-scatter is given below the population gates (middle panel). The gating strategy depicted here was used to isolate CD3^+^CD4^+^MAL^-^ and MAL^+^ T-cells for the biochemical analysis presented in Fig 7.

**
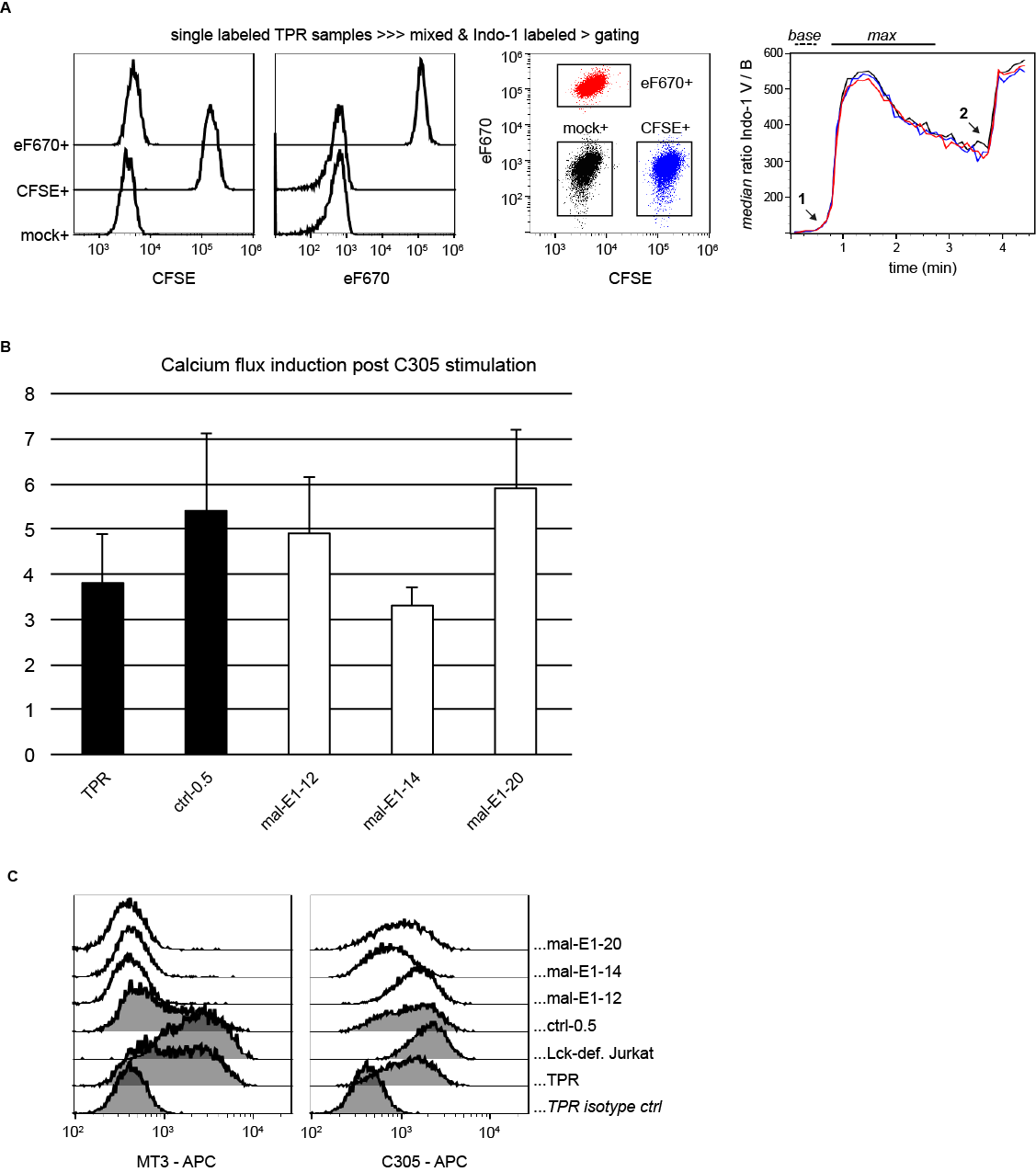
Suppl. Fig.6.**

(A) Calcium flux pre-labelling and maximum induction analysis: To exclude handling-induced variations in Indo-1 labelling and stimulation between different samples, ctrl- and MAL-ko clones were separately CFSE-, eF670- or mock-labelled on the day before calcium flux analysis. Shown exemplarily for TPR cells, labeling efficiency was 100% with zero unlabelled cells in single-labelled samples (left plots) and no double-positive events after mixing of mock-, CFSE- and eF670-labelled cells prior to Indo-1 labeling and flux analysis (middle plot). Gating on separate populations according to pre-labeling for Indo-1 violet/blue ratio evaluation (right plot) demonstrated that different pre-labeling did not affect calcium flux read-out of TPR cells. Induction after mAb C305 stimulation was calculated by dividing the mean of V/B ratio during the maximum induction period (max - black line) by the corresponding baseline ratio (base - dotted line).

(B) Maximum calcium flux induction statistics with S.D. (n=3) from all clones analysed is shown. No significant differences were observed between ctrl- and MAL-ko clones.

(C) Flow cytometric analysis after surface staining of ctrl- and MAL clones with anti-MAL MT3 and anti-TCR C305 mAbs is shown. The non-significant, slight differences in calcium flux behaviour and Lck IS recruitment observed between the clones is independent of MAL surface expression and can be explained by the variability in TCR expression, i.e. we observed the lowest calcium flux induction and Lck recruitment in the MAL-ko#14 cells, which displayed the lowest TCR surface expression.

**
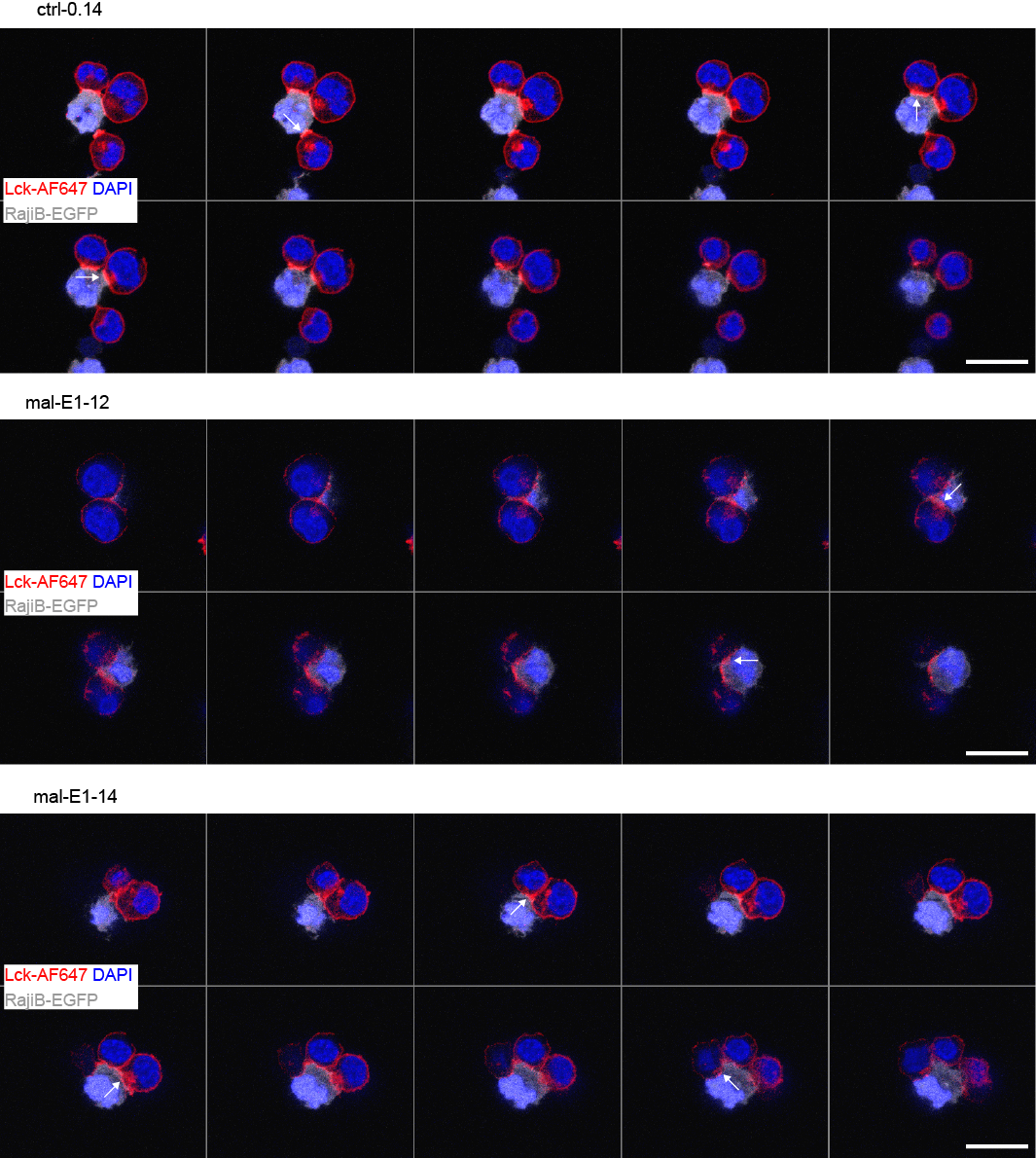
**

**Suppl. Fig. 7.**

Representative z-stacks of confocal analysis after synapse formation and Lck staining are shown for all other TPR clones analysed. Scale bar, 20 µm.

**
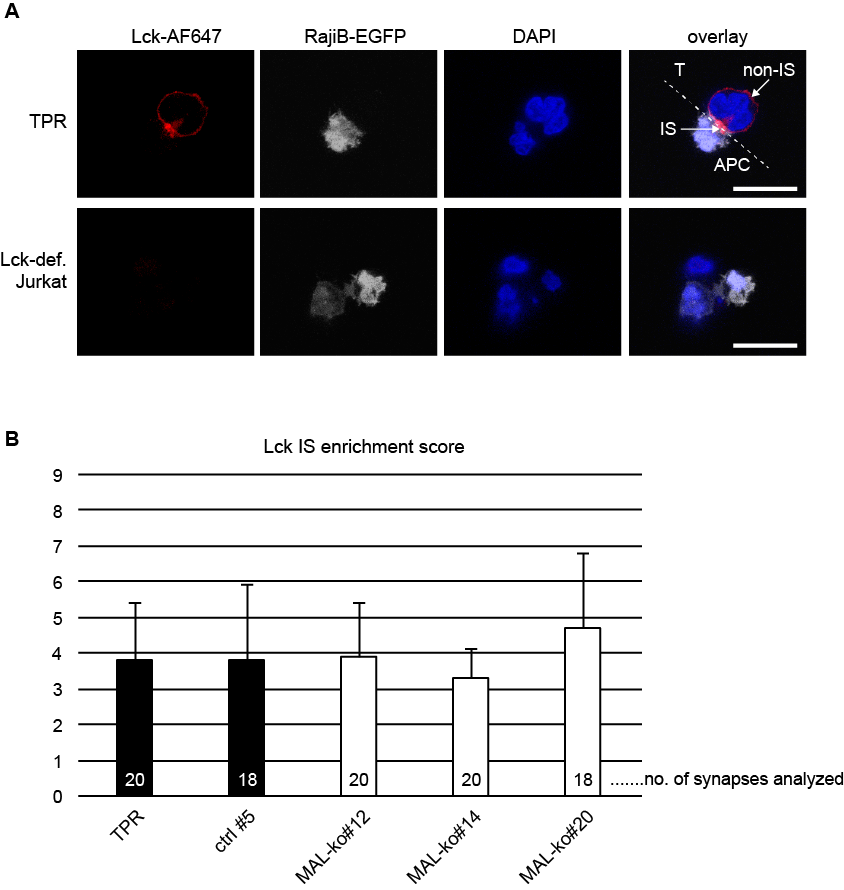
**

**Suppl. Fig. 8.**

(A) Confocal IS analysis: Shown are individual fluorescent channels and the overlay for representative T:APC pairs of parental TPR (positive control) and Lck-deficient Jurkats (negative control), demonstrating Lck recruitment to the IS and specific staining thereof. Lck-AF647 signals at the T:APC-interface/IS and the opposing non-IS membrane site used for Lck enrichment analysis are depicted by arrows. Scale bar, 20 µm.

(B) Lck IS enrichment score analysis: Lck-AF647 intensity profiles at the IS vs. the opposite membrane site (18-20 cells per clone) were analysed using the ZEN2.3 SP1 software Profile tool to calculate the Lck IS enrichment score [= signal at IS / signal at non-IS site]. Lck enrichment at the IS was typically ranging from 3 to 5, with no significant differences observed between all clones analysed.
